# Effects of Carnosine Supplementation on Cognitive Outcomes in Prediabetes and Well-Controlled Type 2 Diabetes: A Randomised Placebo-Controlled Clinical Trial

**DOI:** 10.3390/ph18050630

**Published:** 2025-04-26

**Authors:** Rohit Hariharan, Aya Mousa, Kirthi Menon, Jack Feehan, Barbara Ukropcová, Jozef Ukropec, Martin Schön, Arshad Majid, Giancarlo Aldini, Maximilian de Courten, James Cameron, Simon M. Bell, Barbora de Courten

**Affiliations:** 1 Monash Centre for Health Research Implementation, Faculty of Medicine, Nursing and Health Sciences, Monash University, 43–51 Kanooka Grove, Clayton, VIC 3168, Australia; rohit.hariharan@monash.edu (R.H.); kirthimenon7@gmail.com (K.M.); 2 Department of Medicine, School of Clinical Sciences, Faculty of Medicine, Nursing and Health Sciences, Monash University, 246 Clayton Road, Clayton, VIC 3168, Australia; james.cameron@monash.edu; 3 School of Health and Biomedical Sciences, STEM College, RMIT University, 30 Janefield Drive, Bundoora, VIC 3083, Australia; jack.feehan@rmit.edu.au; 4 Department of Metabolic Disease Research, Institute of Experimental Endocrinology, Biomedical Research Centre, Slovak Academy of Sciences, Dúbravská Cesta 9, 845 05 Bratislava, Slovakia; barbara.ukropcova@gmail.com (B.U.); jozef.ukropec@savba.sk (J.U.); 5 Division of Endocrinology and Diabetology, Medical Faculty and University Hospital Düsseldorf, Heinrich Heine University, Moorenstraße 5 Universitätsklinikum, 40225 Düsseldorf, Germany; martin.schoen@ddz.de; 6 Institute for Clinical Diabetology, German Diabetes Center, Leibniz Center for Diabetes Research at Heinrich Heine University, Auf’m Hennekamp 65, 40225 Düsseldorf, Germany; 7 German Center for Diabetes Research (DZD), Ingolstädter Landstraße 1, 85764 Neuherberg, Germany; 8 Sheffield Institute for Translational Neuroscience, School of Medicine and Population Health, University of Sheffield, 385a Glossop Rd, Sheffield S10 2HQ, UK; arshad.majid@sheffield.ac.uk (A.M.); s.m.bell@sheffield.ac.uk (S.M.B.); 9 Department of Pharmaceutical Sciences, University of Milan, Via Luigi Mangiagalli, 25, 20133 Milan, Italy; giancarlo.aldini@unimi.it; 10 Institute of Health and Sport, Victoria University, 70/104 Ballarat Rd, Footscray, VIC 3011, Australia; maximilian.decourten@vu.edu.au; 11 NIHR Sheffield Biomedical Research Centre, University of Sheffield and Sheffield Teaching Hospitals NHS Foundation Trust, Glossop Rd, Broomhall, Sheffield S10 2JF, UK; 12 Neuroscience Institute, University of Sheffield, Firth Court, 385a Glossop Rd, Broomhall, Sheffield S10 2TN, UK

**Keywords:** carnosine supplementation, cognition and diabetes, cognitive function, prediabetes and cognition, type 2 diabetes, neurocognitive outcomes, randomised controlled trial (RCT), neuropsychological assessment, metabolic health and cognition, diabetes and neurodegeneration, antioxidants and cognitive function, insulin resistance and cognition, CANTAB cognitive testing, executive function and diabetes, inflammation and cognitive decline

## Abstract

**Background:** Trends in global ageing underscore the rising burden of age-related cognitive decline and concomitant cardiometabolic diseases, including type 2 diabetes mellitus (T2DM). Carnosine, a naturally occurring dipeptide with anti-inflammatory, antioxidant and anti-glycating properties, has shown promise in animal models and limited human studies for improving cognitive function, insulin resistance and T2DM, but its therapeutic effects on cognition remain unclear. The aim of this study is to assess the effects of carnosine on cognitive function in individuals with prediabetes or well-controlled T2DM. **Methods:** This is a secondary analysis of a double-blind randomised controlled trial (RCT), whereby 49 adults with prediabetes or early-stage well-controlled T2DM were randomised to receive 2 g of carnosine or identical placebo daily for 14 weeks. At baseline and follow-up, cognitive function was assessed as a secondary outcome using the Digit-Symbol Substitution Test, Stroop test, Trail Making Tests A & B, and the Cambridge Automated Neuropsychological Test Battery (CANTAB). **Results:** In total, 42 adults (23 males and 19 females) completed the trial. There were no differences in participant anthropometry or cognitive functioning between carnosine and placebo groups at baseline (all *p* > 0.1). After the 14-week supplementation period, there were no differences between carnosine and placebo groups in change and follow-up values for any cognitive measures including Stroop, Digit Symbol Substitution Sest, Trail Making A/B or CANTAB (all *p* > 0.05). Adjustments for baseline cognitive scores, diabetic status, level of education, age or interaction effects with participants’ sex did not change the results. **Conclusions:** Carnosine supplementation did not improve cognitive measures in individuals with prediabetes or T2DM in this study. While larger trials may provide further insights, alternative factors—such as the relatively young and healthy profile of our cohort—may have contributed to the lack of observed effect. Future research should examine individuals with existing cognitive impairment or those at higher risk of cognitive decline to better define the therapeutic potential of carnosine in this context.

## 1. Introduction

Type 2 diabetes (T2DM) is a major contributor to global mortality and morbidity and has steadily increased in prevalence over the past decade, from 366 million individuals in 2011 to 536.6 million individuals living with T2DM globally in 2021 [1,2]. These estimates are projected to rise to 783.3 million (11.2% of the global population) by 2045 [2]. Obesity and overweight are key risk factors for T2DM [3], caused by an imbalance between energy consumption and energy expenditure. This imbalance is often driven by a combination of sedentary lifestyles and excessive consumption of highly processed, energy-dense and nutrient-deficient diets, which are often more palatable and less expensive than healthier diets [4]. As of 2022, there were 2.5 billion adults with overweight or obesity, representing a staggering 43% of the global population and an increase from 25% in 1990 [5].

Both obesity and T2DM contribute to neurodegenerative diseases, including Alzheimer’s dementia [6] and Parkinson’s disease [7]. Insulin resistance, the central pathophysiological mechanism of T2DM and a common feature in obesity [8] has been identified as an important risk factor for cognitive impairment [9,10]. Ageing compounds the risk of cognitive impairment, with older individuals with T2DM more likely to have cognitive impairment compared with younger populations. However, interconnected mechanisms underpinning these cognitive changes can promote the development of early cognitive decline even in younger individuals with obesity and T2DM [11,12,13]. In the clinical setting, T2DM and cognitive impairment are treated independently [14], an approach that may be insufficient to address the syndromic nature of either condition. Therefore, investigating new treatments that can act synergistically to improve both neurocognitive function and glucose metabolism in T2DM and at-risk populations is warranted.

Carnosine, a dipeptide consisting of beta-alanine and L-histidine, has well-documented anti-glycating, anti-inflammatory and antioxidant abilities [15], processes which impact both glycaemia and cognitive function [16]. Carnosine is naturally abundant in skeletal muscle tissue and brain tissue in vivo [15] and can be derived through diet, mainly via the consumption of beef, chicken and turkey, as well as prawn, mackerel and tuna meat [17]. Carnosine is also available in supplements [18], where it has been shown to improve exercise performance by boosting the buffering capacity of skeletal muscle and delaying fatigue during intense physical activity [19]. In the context of cognition, the anti-inflammatory properties of carnosine are of particular relevance since inflammation is correlated with increased oxidative stress, and both are associated with low cognitive performance [20]. Indeed, antioxidant and anti-inflammatory agents which reduce oxidative stress and neuroinflammation, including endogenous histidine-containing dipeptides (HCDs) [15], vitamin K2 [21], polyphenols from olive trees [22], isoamericanin A [23], and ellagitannin [24], have been shown to reduce cognitive decline in preclinical models [20].

However, studies examining the effects of carnosine on cognition and metabolism to date have been largely based on animal and experimental models, with limited human data [25]. In our recent meta-analysis, we identified 10 RCTs examining the impact of HCDs on cognitive function, eight of which supplemented carnosine combined with anserine, with none using carnosine alone. Although we reported improved memory in elderly populations and improved delayed recall following supplementation among individuals with and without mild cognitive impairment assessed using the Wechsler Memory Test (WMS-2), these effects could not be attributed solely to carnosine. The same systematic review identified other key gaps in the evidence, namely the reliance on relatively brief global tests of cognition following carnosine supplementation [16,26]. Additionally, no studies have examined whether carnosine supplementation may affect cognitive measures in prediabetes or T2DM, despite the reported neurocognitive benefits of carnosine and the higher prevalence of cognitive impairment—often uncurable, in T2DM [27].

To address this critical evidence gap, the aim of the present study was to examine the effects of carnosine supplementation on cognitive function using RCT data with comprehensive neuropsychological and cognitive assessments in individuals with prediabetes or early-stage well-controlled T2DM.

## 2. Results

**Figure 1:**
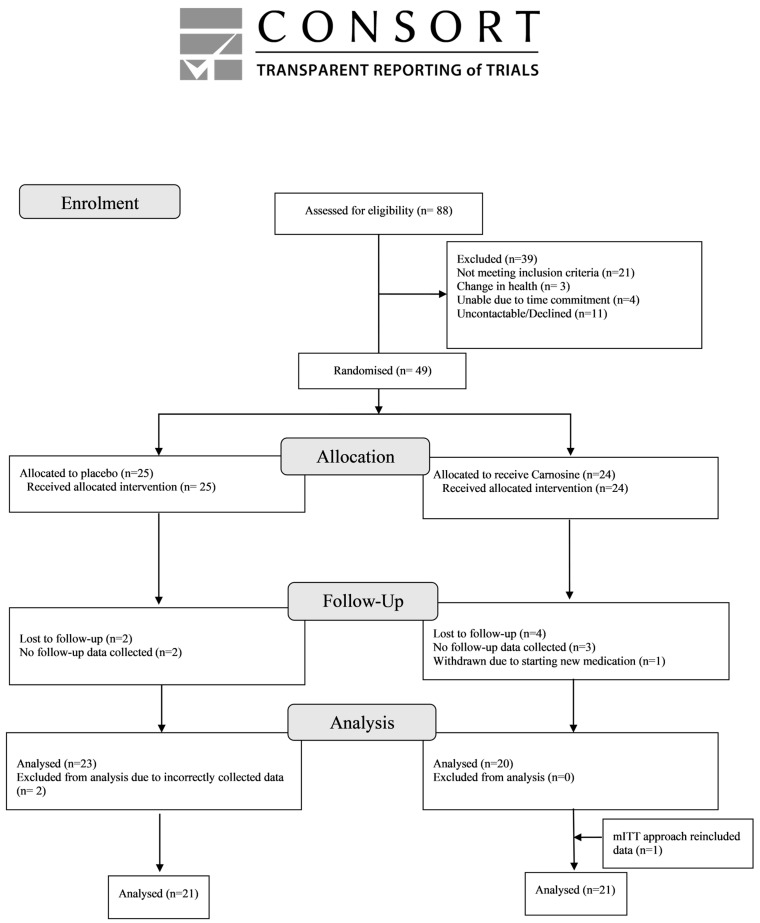
CONSORT flow diagram.

A total of 49 participants were randomised, with 24 assigned to the carnosine intervention (1 g orally twice daily) and 25 to the placebo group. Baseline characteristics did not differ between groups (Table 1 and Table S1). The modified intention-to-treat (mITT) analysis included all participants with a follow-up assessment. A total of seven participants were excluded from the analysis: five due to the absence of follow-up data and two due to incorrectly collected cognitive data. Consequently, 42 participants (21 per group) were included in the final mITT analysis (Figure 1). Data were collected between 2016 and 2020.

Among these 42 participants, the mean age was 52.0 ± 10.2 years (range 28–66 years), and 19 were female. There were 25 (60%) individuals with prediabetes, while the remaining 17 (40%) had T2DM and were taking metformin. All participants were cognitively healthy, as those with any history of neurological conditions would have been excluded from the trial. The participants reported good tolerance to carnosine, with none reporting any side effects.

### Cognitive Performance Outcomes

For the Digit Symbol Substitution Test (DSST) (Table 2 and Supplementary Section S2), change values were not statistically different between groups (*p* = 0.46; Table 2). Multivariable regression analyses (Table 3) adjusting for baseline DSST scores, diabetic status, education and age also showed no significant effects (all *p* ≥ 0.20; Table 3), and there were no interactions by sex (*p* = 0.96). Subgroup analyses also did not show significant differences between carnosine and placebo groups (all *p* > 0.30) (Tables S2.1, S2.2, S2.3 and S2.4).

Stroop Test results (Table 2, Table 3 and Table S3 and Supplementary Section S2) showed no significant differences in change values for “Off Time” (*p* = 0.75), “Off Time + On Time” (*p* = 0.99), or interference state “On time–Off time” (*p* ≥ 0.68) between groups. Multivariable regression models adjusted for baseline Stroop scores, diabetic status, education level and age did not alter results (all *p* > 0.80) (Table 3, Tables S3.1 and S3.2), and there were no interaction effects by sex (*p* > 0.80). Subgroup analyses (Table S3.3) comparing participants with prediabetes to those with T2DM (Tables S3.3.1, S3.3.2, S3.3.3 and S3.3.4) also showed no significant results (all *p* ≥ 0.40).

For the Trail Making Test (TMT-A & B) (Table 2 and Supplementary Section S2), there were no significant differences in change values for TMT-A (*p* = 0.76), TMT-B (*p* = 0.42), or TMT-B:A ratio (*p* = 0.66) between carnosine and placebo groups. These results remained unchanged after adjustment for baseline scores, diabetic status, education and age (all *p* ≥ 0.20) and in the interaction analyses (*p* > 0.20, as well as in subgroup analyses (all *p* > 0.40) (Tables S4.1, S4.2, S4.3 and S4.4).

In the CANTAB assessments (Table 2, Table 3 and Table S5.1), no significant differences were found across any test domains. For the Delayed Match to Sample (DMS) test, key variables such as percentage correct, median latency and probability of error showed no group differences in change values (*p* ≥ 0.46), including in adjusted analyses (Table 3) and interaction effects with sex (*p* > 0.31). Similarly, in the Paired Associates Learning test (Table 3), there were no significant differences in measures such as first-attempt memory score, total errors, mean errors to success or total attempts, including after adjustment for covariates (*p* ≥ 0.18). There were no interactions by sex (*p* > 0.36), and multivariable subgroup analyses comparing prediabetes and T2DM groups also yielded no significant findings (all *p* > 0.18; Tables S5.2.1, S5.2.2, S5.2.3 and S5.2.4) were observed.

Changes in Pattern Recognition Memory (PRM), including percent correct latency or median correct latency, did not significantly differ between groups in either immediate or delayed measures (Table 2). This result remained consistent across univariable, multivariable, interaction and subgroup analyses (*p* ≥ 0.40; Table 2, Table 3, Tables S5.2.1, S5.2.2, S5.2.3 and S5.2.4). Likewise, for the Reaction Time Index (RTI) and the Rapid Visual Processing (RVP) domains (Table 2 and Table 3), the latter including key variables such as median response latency and probability of false alarms, there were no significant changes detected between groups in univariable, adjusted or subgroup analyses (all *p* > 0.46) or in interaction analyses by sex (*p* > 0.53).

Lastly, in the Spatial Working Memory (SWM) domain, measures such as between errors and strategy scores showed no significant differences between carnosine and placebo groups (Table 2 and Table 3). Results were unchanged in multivariable regression models (*p* ≥ 0.31), subgroup analyses (all *p* ≥ 0.83) (Tables S5.2.2 and S5.2.4) and interaction analysis by sex (*p* > 0.50).

Carnosine was well tolerated, with no adverse effects linked to the supplementation. The sole reported concern was minor pain and bruise at the blood draw site experienced by one participant.

## 3. Discussion

To our knowledge, this is the first double-blind RCT investigating the effects of carnosine supplementation on cognitive function in individuals with prediabetes or T2DM. Our findings showed that short-term carnosine supplementation had no significant effect on cognitive function despite the use of comprehensive neuropsychological assessments, including the CANTAB series of computerised cognitive tests. These findings remained unchanged after controlling for key factors known to influence cognition, including diabetic status, age, sex and level of education, suggesting that carnosine supplementation may not provide cognitive benefits in this population.

No differences were observed between the carnosine and placebo groups across cognitive tests assessing executive function, attention, cognitive flexibility and processing speed. In particular, the Paired Associates Learning measures of the CANTAB series, which are highly sensitive to mild cognitive impairment (MCI) and early Alzheimer’s pathology [28,29,30], were unchanged by carnosine supplementation, including after adjustment for covariates. One possible explanation for the lack of observed effects is that participants in this study had no known pre-existing cognitive impairment, potentially limiting the ability to detect measurable cognitive benefits. However, three previous studies in participants who were similarly healthy or had MCI have reported conflicting findings, showing that orally ingested carnosine improved cognitive performance after 12 weeks [31,32,33]. These trials involved older participants (mean age over 60 years) who were also more homogeneous (all recruited from the Tokyo metropolitan area), in contrast to the present RCT, which included a younger and more ethnically diverse cohort, including Caucasian, South American, Middle Eastern, South Asian and Southeast Asian participants. Additionally, although the average dose of carnosine used was lower than in the current study (ranging from 250 mg to 1 g daily compared with 2 g daily), two studies administered a combination of carnosine and anserine, making it difficult to isolate the specific effects of carnosine.

Importantly, previous RCTs investigating the effects of carnosine on cognition [31,32,33,34,35,36], including those noted above, have been constrained by limited numbers and types of neuropsychological testing, typically using assessments such as the Mini Mental State Examination (MMSE), Alzheimer’s Disease Assessment Scale (ADAS), Clinical Dementia Rating (CDR), WMS (Wechsler Memory Scale), Short Test of Mental Status (STMS) or AVLT (Auditory Verbal Learning Test). While these tools are efficient in screening for specific cognitive deficits, none except the MMSE can derive a global assessment of cognitive functioning, and most lack the granularity to detect subtle changes in cognitive functioning. This contrasts with the domain-specific approach used in the current study, which is the first RCT to employ a comprehensive battery of objective cognitive assessments—including the gold-standard CANTAB, Stroop, DSST, and Trail Making Tests—to evaluate the cognitive effects of carnosine in prediabetes or T2DM. This specific test combination was chosen for its ability to assess diverse memory domains and complex cognitive functions, providing a more detailed analysis of cognitive performance. The use of computerised tests such as the CANTAB also minimises ceiling effects, enabling greater sensitivity in detecting subtle cognitive changes. Moreover, the more detailed statistical analysis used in this study suggests that many of the effects of carnosine on cognition reported previously may be confounded by variables known to affect cognition and/or potentially erroneous data due to multiple tests.

Despite the absence of an effect in this study, a potential effect of carnosine on neurocognitive function remains plausible from a mechanistic perspective. Recent findings from the NEAT trail [37] indicated cognitive benefits in younger healthy adults (23–35 years), suggesting age-dependent effects. The NEAT trial [37] included a larger and healthy cohort, whereas the participants from our trial had metabolic conditions, such as impaired glucose metabolism, that could influence cognitive outcomes, particularly in those with prediabetes and T2DM. Increased levels of fasting plasma glucose, 2 h postprandial glucose, HbA1c and fasting plasma insulin are important determinants in the development of cognitive impairment [38], especially in carriers of the APOE4 gene [39]. A meta-analysis of 122 studies reported a significant increase in the risk of developing cognitive disorders and all-cause dementia, Alzheimer’s disease and vascular dementia in those with T2DM and prediabetes [38]. Carnosine has the ability to improve glucose metabolism in those with prediabetes and T2DM, as shown by our group previously [40,41]. Further, carnosine, along with anserine, has the ability to cross the blood–brain barrier (BBB) and induce the expression of neurotrophic genes in human neuronal cells and in glial cells, which produce brain-derived neurotrophic factor (BDNF) and neuronal growth factor (NGF) in the brain [42]. However, there is limited evidence of carnosine’s penetrance of the BBB into neural tissues in vivo in humans. Carnosine is rapidly hydrolysed by the enzyme carnosinase to its constituents in the gut, limiting its ability to saturate distal tissue sites, and this may explain in part the lack of significant differences in this study. Future research aiming to elucidate the true pharmacodynamics of carnosine in human neural tissues should utilise phosphate MR-spectroscopy to image changes in metabolite concentrations and fractional volumes of brain grey and white matter post-supplementation [43,44].

The primary limitation of this study is its small sample size, as it is a secondary analysis of an RCT originally designed to examine the effects of carnosine on glycaemic parameters rather than on cognitive performance. This study was, therefore, likely underpowered to detect the smaller effect sizes typically seen in cognitive research. A post hoc power analysis suggests that future trials would require much larger sample sizes, with approximately 350 participants per group, to achieve 90% power to detect the small effect sizes commonly seen in cognitive outcome studies. Additionally, our study population did not include participants with cognitive dysfunction, limiting the generalisability of our results to these populations. Participants had relatively well-controlled diabetes (HbA1c < 8%) and typically shorter durations since diagnosis of T2DM (2 months to 6 years). Evidence suggests that cognitive impairment in prediabetes and T2DM is minor in those with shorter duration of diabetes compared with those with long-standing chronic diabetes (>13 years) [45], suggesting that supplementation-induced changes may be more detectable in populations with longer disease durations and concomitant cognitive impairment. The dosage (2 g) and duration (14 weeks) of carnosine supplementation used in this trial may also have been insufficient to affect cognitive measures to an extent detectable within the constraints of the sample size and statistical power. Given that longer durations (at least 24 months) have been recommended for interventional studies of cognitive function in Alzheimer’s disease and MCI [46], future studies should consider extended follow-up periods to assess cumulative cognitive effects. Finally, the average age of participants was 54 years, and it is possible that carnosine is more effective in older populations with early indications of cognitive decline. This is supported by a previous meta-analysis [47] by our group, which found that carnosine supplementation improved delayed recall in older groups (>65 years) but not in younger cohorts.

Notwithstanding these limitations, this is the first RCT examining carnosine supplementation for cognitive function in prediabetes and T2DM. We utilised comprehensive neuropsychological testing in a well-characterised cohort, albeit with a likely smaller sample size than required to detect significant differences. While the cognitive benefits of carnosine could not be demonstrated in the present study, potential effects cannot be ruled out, and our data generate important insights to advance this area of study. Future trials should aim to recruit larger sample sizes with diverse baseline risk profiles, age groups and ethnicities. Key factors such as physical activity levels, pre-existing cognitive impairment, T2DM durations and diagnosis of T2DM using American Diabetes Association (ADA) guidelines, which incorporate HbA1c [48], should be carefully considered to ensure a more representative study population. Trials should also incorporate domain-specific neuropsychological testing to improve internal and external validity and clarify the effects of carnosine, if any, on cognitive performance in high-risk populations.

## 4. Materials and Methods

### 4.1. Study Design and Participants

This is a secondary analysis of a double-blind placebo-controlled RCT investigating the effects of 14 weeks of carnosine supplementation on glycaemic control in individuals with prediabetes or T2DM, the primary results of which are published elsewhere [41]. Detailed methodology is provided in our published protocol [49]. Briefly, we included participants aged 18–70 years with prediabetes or T2DM diagnosed using oral glucose tolerance test (OGTT) (as per World Health Organization [WHO] guidelines [50,51]) and who were diet controlled and not taking any medications except metformin, which participants continued taking during the trial. Participants did not undergo cognitive testing prior to recruitment and were assumed to be cognitively healthy based on the history they provided. Those with haemoglobin A1c (HbA1c) higher than 8% at the time of screening were excluded. Furthermore, participants with a body mass index (BMI) > 40 kg/m^2^; fluctuating body weight (>5 kg weight change in the prior 12 months); smoking, or alcohol consumption [52] (standard drinks > 10 per week or >4/day); concomitant central nervous system, cardiovascular, respiratory, haematological or gastrointestinal diseases; and women who were pregnant or lactating were also excluded. Participants were instructed not to alter their diet or physical activity during the trial.

### 4.2. Recruitment

Primary advertising for the trial was conducted via newspapers, radio and posters. Volunteers were screened via a telephone-based questionnaire and, if eligible, were invited to the Clinical Trials Centre of the Monash Health Translational Research Precinct for an in-person screening visit. At the screening visit, prospective participants underwent a 75 g OGTT to confirm prediabetes/T2DM status using WHO (2006) criteria [50] and blood tests to exclude other underlying conditions. All participants were assessed against the inclusion and exclusion criteria noted above prior to formal inclusion and randomisation. Participants were free to withdraw at any time they desired and were encouraged to speak to the study physician. They were informed that all personal data would be deidentified, with only clinical data retained.

### 4.3. Ethics

All participants provided written informed consent before attending the baseline screening investigations. The trial was registered on clinicaltrials.gov (NCT02917928, 28/09/16) and conducted as per the published protocol [49] in line with the Standardised Protocol Interventions: Recommendations for Interventional Trials [53], with reporting conforming to the CONSORT guidelines [54]. The Monash Health Human Research Ethics Committee provided ethics approval for the trial (Ref: 16061A), and all trial processes complied with the Declaration of Helsinki [55].

### 4.4. Intervention and Randomisation

Computer-generated randomisation codes were provided by the study statistician using block sizes of four by sex, which were then forwarded to the clinical trials pharmacist. Participants were randomised to 2 g of oral carnosine (1 g twice daily, CarnoPure™ Flamma S.p.A, Chignolo d’Isola (BG), Italy), including on the clinical testing days, or an equivalent placebo group receiving 2 g of oral methylcellulose (1 g twice daily; Pharmaceutical Packaging Pty Ltd., Melbourne, Australia). Both groups were instructed to take the assigned intervention for 14 weeks. The sample size, intervention dosages and duration utilised were decided based on a pilot study and previous meta-analysis conducted by our group [40,56]. To ensure double-blinding of participants and researchers, both carnosine and placebo were provided in identical capsules within indistinguishable 60-capsule containers. All participants and trial personnel, including study physicians, nurses and research assistants, remained blinded to the intervention allocation assigned by the trial pharmacists until after trial closure and primary analyses were complete. The carnosine and the placebo powder were assessed for quality and crystallinity by an independent chemical laboratory and deemed to be free of impurities. Carnosine supplementation at these dosages has been used safely in previous studies with no reported side effects or toxicity. During informed consent, participants were advised that should any side effects arise, such as light-headedness, nausea or transient sensations of feeling hot, they should immediately inform the study physician. Treatment compliance was self-reported by participants to the study physicians. Compliance was assessed using the return of containers and counting of remaining capsules at the last follow-up visit.

### 4.5. Outcome Measures

The primary outcome of the RCT was a change in 2 h glucose levels after 14 weeks of carnosine supplementation, with results reported elsewhere showing beneficial effects [28]. This study examined secondary cognitive outcomes assessed in English through four tests in written and digital formats, detailed in Supplementary S1 and includes the following: (1) The Digit Symbol Substitution Test, performed in a paper format using a pre-printed questionnaire. The instruction set displayed at the top of the questionnaire had a row of numbers from ‘1 to 9’ with corresponding non-language symbols to assess cognitive flexibility, attention and executive function; (2) The Stroop test was administered via the Encephalapp (www.encephalapp.com) application [57] and is intended to measure cognitive flexibility and processing speed; (3) The Trail Making Tests (A&B) were administered using the “Trail Making Test UK/XIF5/1014/0071” application (Norgine Pharmaceuticals Ltd., Harefield, UK) available during the trial, and assess executive functioning; and (4) The Cambridge Neuropsychological Automated Battery (CANTAB) tests were conducted on a digital tablet (iPad iOS v.10) to collect several metrics of cognitive function through different tasks.

For the CANTAB, we tested six domains as follows: (i) delayed match to sample to test the cognitive domains of attention and short-term visual recognition memory [58]; (ii) paired associates learning to test visual episodic memory [58] via a multi-stage process of remembering patterns and locations of patterns; (iii) pattern recognition memory to assess visual recognition memory [58], where participants select a previously seen pattern after being shown a series of visually complex patterns in a continuous sequence (iv); reaction time index to examine the domains of processing and psychomotor speed [58]; (v) rapid visual processing to test sustained attention, administered as a random sequence of numbers ticking in a white box in the centre of the screen, and participants press a button when the target sequence appears; and (vi) spatial working memory which assesses working memory and strategy [58] by measuring the ability to process and store spatial memory and apply it in answering the test questions.

All participants were tested at baseline (prior to randomisation) and after 14 weeks of supplementation (follow-up). Participants were contacted at week 4 and week 10 via phone to enquire about treatment side effects and compliance. Testing was conducted in the same location but not at the same time of day, and participants received a brief explanation of the method for each test before commencing their attempt. Participants were not fasting during testing and were seated in a relaxed, bright and controlled setting to limit external stimuli (noise, distractions), with only the participant and researcher present in the room.

### 4.6. Statistical Analysis

For this analysis, data were analysed using JASP (v0.18.3, University of Amsterdam) and SPSS (v29, IBM Corp, Armonk, NY, USA). Initially, a complete-case analysis was considered, but Little’s MCAR (missing completely at random) test indicated that some data were missing at random (MAR), with no variable exceeding 10% missing data. Consequently, a modified intention-to-treat (mITT) approach was applied, including all randomised participants who completed at least one follow-up cognitive assessment after initiating treatment. Participants lost to follow-up before contributing cognitive follow-up data were excluded, while those who withdrew post-randomisation but had follow-up data were retained. Additionally, participants with incorrectly collected cognitive data were excluded.

Baseline characteristics (Table 1) were compared using independent-sample *t*-tests. A power analysis confirmed 80% power to detect effect sizes between 0.91 and 1.17, whereas no cognitive variable exceeded an effect size of 0.61. Missing data were imputed using predictive mean matching with five imputations. Sensitivity analysis compared mITT and imputed datasets by assessing pooled estimates from multiple imputations using Rubin’s rules to evaluate whether imputation significantly altered results.

Cognitive variables were assessed for normality using histograms and the Shapiro–Wilk test. Descriptive statistics were reported as means with standard deviations (SDs) or medians with interquartile ranges (IQRs) for non-normal data. Change (delta) values were calculated by subtracting follow-up from baseline values, with between-group differences analysed via independent samples *t*-tests. Effect sizes were estimated using Cohen’s δ.

A general linear model (GLM) was fitted to both original and imputed datasets, yielding pooled mean estimates for each variable while controlling for baseline values, intervention group (placebo; carnosine), diabetes status (prediabetes; T2DM), education level (primary; secondary; tertiary and above) and age. Exploratory analyses included an interaction term between sex and intervention group and subgroup analyses for participants with prediabetes or T2DM.

Following univariable analysis, delta values were included in multivariable linear regression models. The first model adjusted for baseline cognitive values, while subsequent models incorporated adjustments for diabetes status (Model 2), education (Model 3), age (Model 4), and sex-by-intervention interaction (Model 5). For the CANTAB test, key outcome variables were selected based on CANTAB guidelines [34], presented in Table 2 and Table 3, with additional variables in the Supplementary Materials. To correct for multiple comparisons, *p*-values were adjusted using the Benjamini–Hochberg procedure [59,60]. Adjusted *p*-values were used to determine statistical significance at a two-tailed threshold of *p* < 0.05.

## 5. Conclusions

In this double-blind, placebo-controlled RCT, carnosine supplementation did not improve cognitive function in individuals with prediabetes or well-controlled T2DM compared with placebo. While this study was unable to detect potentially subtle cognitive effects, it highlights key considerations for future research, namely the need for larger, longer-duration trials with comprehensive neuropsychological testing. Such data will help establish more definitively whether carnosine can mitigate the cognitive impairment commonly seen in metabolic disorders such as T2DM.

## Figures and Tables

**Table 1:** Sample characteristics at baseline.

Variable	Placebo (21)	Carnosine (21)	
	Mean ± SD	Mean ± SD	
Demographics			
Age	50.17 ± 10.64	51.7 ± 10.19	
Sex (males), *n* (%)	15 (75%)	14 (67%)	
Prediabetes/diabetes proportions, *n* (%)	15 (71%)/6 (29%)	10 (47.6%)/11 (52.4%)	
Metformin: yes/no, *n* (%)	9 (43%)/12 (57%)	8 (38%)/13 (62%)	
Digit symbol test			Performance Range
Score: (Number of correct responses)	70.5 ± 9.987	64.89 ± 17.62	1–93
Stroop tests			
Off Time	66.81 ± 7.9	70.14 ± 13.62	1–125.84
Off Time + On Time	142.62 ± 15.48	153.52 ± 28.9	1–274.9
On Time	75.81 ± 8.74	83.38 ± 16.19	1–148.7
On Time–Off Time	9 ± 6.19	13.25 ± 7.73	1–29.8
Successful times ×attempts (Off)	380.31 ± 74.61	389.67 ± 97.76	NA
Successful times × attempts (On)	440 ± 112.74	470.05 ± 98.02	NA
Trail making Tests			
Trail making Test A (s)	17.05 ± 5.13	16.75 ± 4.90	1–300
Trail making Test B (s)	58.38 ± 31.07	63.18 ± 39.56	1–300
TMT ratio (B/A)	3.59 ± 1.6	3.76 ± 1.90	NA
Cambridge Neuropsychological Test Automated Battery			
Delayed Match to Sample (DMS)			
DMS Percent correct (all delays)	87.00 (13.00)	87.00 (13.00)	0–100
DMS Probability of error given correct	0.12 (0.12)	0.12 (0.13)	−1 to 1
DMS Probability of error given error	0 (0)	0 (0)	−1 to 1
DMS Total correct (simultaneous)	18.00 (2.00)	18.00 (2.00)	0–20
Paired Associates Learning (PAL)			
PAL First-attempt memory score	12.20 (3.00)	12.70 (4.05)	0–20
PAL Mean errors to success	2.05 (1.43)	1.50 (1.54)	0–4
PAL Number of patterns reached	8.00 (0)	8.00 (0)	2–8
PAL Total attempts	8.00 (1.50)	7.50 (3.00)	0–4
PAL Total errors	12.00 (11.00)	13.00 (9.00)	0–80
PAL Total errors (adjusted)	15.00 (13.00)	14.50 (16.75)	0–70
Pattern Recognition Memory (PRM)			
Pattern Recognition Memory Median correct latency–Delayed	1978.25 (458.13)	1778.75 (390.25)	100–∞
Pattern Recognition Memory median correct latency–Immediate	1618.25 (281.75)	1612.00 (440)	100–∞
PRM Percent Correct Delayed	100 (8.33)	91.67 (8.34)	0–100
PRM Percent Correct Immediate	95.84 (8.33)	100 (8.33)	0–100
Reaction time index (RTI)			
RTI Five-choice error score (all)	0 (0)	0 (1.00)	0–30
RTI Simple error score (all)	2.00 (3.00)	1.00 (2.00)	0–30
RTI Simple movement time	203.00 (107.50)	193.50 (42.50)	100–5100
RTI Simple reaction time	39.24 (25.45)	38.20 (23.76)	100–5100
Rapid visual processing (RVP)			
RVP A Prime	0.89 (0.06)	0.91 (0.06)	0–1
RVP Response latency	192.66 (81.68)	149.65 (96.14)	100–1900
RVP Total false alarms	5.00 (8.00)	1.00 (3.00)	0–546
RVP Total hits	31.00 (10.25)	36.00 (13.00)	0–54
RVP Total misses	23.00 (10.25)	18.00 (13.00)	0–54
Spatial working memory (SWM)			
SWM Between errors	10 (21.25)	12.00 (11.00)	0–90
SWM Strategy	8.00 (6.00)	8.00 (4.00)	2–14

Results are presented as mean ± standard deviation or median (interquartile range) unless otherwise specified.

**Table 2:** Cognitive test scores between carnosine supplementation and placebo.

Test Variable	Placebo			Carnosine			*p* *	*p*(adj)
	Baseline	Follow-Up	Δ	Baseline	Follow-Up	Δ		
Digit Symbol Test								
Score	71.37 ± 10.58	75.67 ± 10.71	3.67 ± 9.48	65.47 ± 17.85	76.00 ± 13.95	10.53 ± 12.90	0.08	0.46
Trail Making test								
Trail Making Test A (s)	17.05 ± 5.13	17.36 ± 5.00	0.31 ± 6.2	16.75 ± 4.90	15.67 ± 4.85	−1.09 ± 5.72	0.46	0.76
Trail Making Test B (seconds)	58.38 ± 31.07	61.71 ± 36.82	2.26 ± 26.0	63.18 ± 39.56	46.84 ± 10.95	−16.34 ± 42.35	0.1	0.42
B:A (s)	3.59 ± 1.6	3.66 ± 2.1	−0.01 ± 1.55	3.76 ± 1.90	3.13 ± 0.80	−0.63 ± 1.86	0.26	0.66
Stroop								
Off Time (s)	66.81 ± 7.90	64.22 ± 10.44	−2.45 ± 12.87	70.14 ± 13.62	72.24 ± 10.01	0.56 ± 7.84	0.05	0.75
On Time(s)	75.81 ± 8.74	81.99 ± 19.41	6.18 ± 17.81	83.38 ± 16.19	90.05 ± 22.06	6.66 ± 20.28	0.29	0.67
Off Time + On Time(s)	142.62 ± 15.48	166.19 ± 97.22	23.57 ± 97.17	153.52 ± 28.90	184.49 ± 79.96	30.97 ± 83.10	0.62	0.84
On time—Off time(s)	9.00 ± 6.19	13.79 ± 14.17	4.4 ± 14.34	13.25 ± 7.73	11.73 ± 4.52	−1.12 ± 7.53	0.28	0.68
Successful times x attempts (Off)	380.31 ± 74.61	361.14 ± 107.19	−12.33 ± 116.04	389.67 ± 97.76	399.54 ± 70.46	−3.81 ± 74.89	0.29	0.64
Successful times x attempts (On)	439.99 ± 112.74	458.21 ± 100.92	12.97 ± 117.89	470.05 ± 98.02	505.21 ± 128.93	28.65 ± 88.31	0.57	0.85
CANTAB								
DMS Percent Correct (All Delays)	84.7 ± 9.00	87 ± 10.76	2.60 ± 14.32	84.10 ± 13.70	85 ± 8.86	0.90 ± 17.81	0.74	0.70
DMS percent correct (Simultaneous)	88.25 ± 6.74	89.52 ± 8.05	1.75 ± 10.29	86.91 ± 11.67	87.38 ± 7.35	0.48 ± 15.08	0.70	0.85
DMS median correct latency	3569.20 ± 914.96	3459.86 ± 1235.52	−97.75 ± 1109.50	3427.29 ± 1747	3248.98 ± 1548.87	−178.31 ± 1467.79	0.84	0.88
DMS Probability of error given error	0.02 ± 0.90	0.09 ± 0.16	0.06 ± 0.20	0.07 ± 0.19	0.10 ± 0.15	0.03 ± 0.27	0.68	0.85
PALFAMS (Paired Associates Learning First attempt memory score)	12.20 ± 3.00	14.32 ± 3.92	2.11 ± 3.25	12.70 ± 4.05	12.40 ± 4.02	−0.37 ± 4.81	0.07	0.54
PALMETS (Paired Associates Learning Mean Errors to Success)	2.05 ± 1.43	1.24 ± 1.00	−1.00 ± 1.49	1.50 ± 1.54	2.35 ± 1.35	0.84 ± 1.34	<0.001	0.05
PALTA (Paired Associates Learning Total Attempts)	8.32 ± 1.77	6.86 ± 1.77	−1.53 ± 1.47	7.65 ± 1.90	7.65 ± 1.66	−0.05 ± 2.09	0.02	0.46
PALTA 4 (Paired Associates Learning Total Attempts 4 Patterns)	1.58 ± 0.61	1.24 ± 0.70	−0.32 ± 0.89	1.25 ± 0.72	1.45 ± 0.83	0.21 ± 0.71	0.05	0.59
PALTEA (Paired Associates Learning Total errors (adjusted))	15.40 ± 10.66	12.37 ± 14.40	−3.00 ± 9.21	17.95 ± 14.40	13.95 ± 12.60	−4.26 ± 12.94	0.73	0.86
PRMPCD (Pattern Recognition Memory Percent Correct Delayed)	94.91 ± 7.08	93.33 ± 7.66	−0.49 ± 9.07	87.084 ± 11.93	84.53 ± 13.77	−3.33 ± 17.19	0.53	0.84
PRMMDCLD (Pattern Recognition Memory Median correct latency)–Delayed	2065.92 ± 626.63	1814.38 ± 391.45	−255.94 ± 633.16	1900.22 ± 457.70	1968.23 ± 679.11	68.00 ± 766.33	0.17	0.65
PRMMDCLI (Pattern Recognition Memory median correct latency)–Immediate	1633.93 ± 386.86	1540.95 ± 280.27	−94.50 ± 312.68	1617.26 ± 326.25	1570.38 ± 328.71	−46.88 ± 211.89	0.57	0.87
PRMPCI (Pattern Recognition Memory Percent Correct Immediate)	93.34 ± 9.97	96.49 ± 6.40	3.51 ± 11.22	95.24 ± 6.26	94.05 ± 11.83	−1.19 ± 9.96	0.17	0.6
RTIFMMT (Reaction Time Index–mean five choice movement time)	292.09 ± 82.54	267.26 ± 63.07	−24.83 ± 81.86	255.14 ± 86.82	271.17 ± 103.42	16.03 ± 48.68	0.06	0.55
RTIFMDMT (RTI Median Five-Choice Movement Time)	290.38 ± 80.76	268.81 ± 64.88	−21.57 ± 82.07	253.57 ± 86.25	268.95 ± 102.64	15.38 ± 50.00	0.09	0.46
RTIFMDRT (RTI Median Five-Choice Reaction Time)	401.07 ± 46.76	407.50 ± 50.94	6.43 ± 34.95	402.67 ± 41.52	399.24 ± 46.23	−3.43 ± 32.34	0.35	0.64
RTIFMTSD (Reaction Time Index -five choice movement time (SD))	40.31 ± 17.22	38.19 ± 20.97	−2.12 ± 24.70	37.49 ± 14.09	36.86 ± 12.49	−0.63 ± 13.06	0.81	0.87
RTISES (Reaction time Index–Simple error Score)	1.91 ± 2.39	0.33 ± 0.56	−0.24 ± 3.21	2.48 ± 5.45	0.43 ± 1.12	0.29 ± 4.3491	0.66	0.84
RTIMSMT (Reaction Time Index–Mean simple movement time)	232.70 ± 75.09	237.12 ± 58.91	4.42 ± 56.76	207.12 ± 79.63	220.89 ± 75.64	13.77 ± 64.70	0.62	0.86
RTIMSRT (Reaction Time Index–Mean simple reaction time)	353.96 ± 38.80	373.28 ± 41.00	19.32 ± 31.79	350.28 ± 42.60	357.92 ± 40.72	7.64 ± 34.67	0.26	0.75
RTISMDRT (RTI Simple Median Reaction Time)	345.07 ± 36.25	360.90 ± 37.40	15.83 ± 26.95	342.10 ± 41.28	347.67 ± 36.55	5.57 ± 31.62	0.26	0.7
RTISMDMT (RTI Simple Median Movement Time)	228.67 ± 72.67	234.45 ± 57.43	5.79 ± 55.44	205.64 ± 78.83	216.71 ± 72.15	11.07 ± 61.23	0.77	0.84
RVPA (Rapid Visual Processing A prime)	0.88 ± 0.06	0.90 ± 0.39	0.02 ± 0.052	0.91 ± 0.04	0.92 ± 0.06	0.009 ± 0.044	0.35	0.62
RVPMDL (Rapid Visual Processing–median response latency)	493.88 ± 116.66	465.53 ± 88.86	−28.35 ± 69.75	481.91 ± 60.09	472.68 ± 61.38	−11.53 ± 62.04	0.43	0.73
RVPPFA (RVP Probability of False Alarm)	0.04 ± 0.117	0.012 ± 0.008	−0.03 ± 0.12	0.007 ± 0.011	0.01 ± 0.037	0.007 ± 0.028	0.19	0.62
SWMBE (Spatial Working Memory–between errors)	11.80 ± 9.80	12.35 ± 10.16	0.55 ± 9.59	12.33 ± 7.84	9.43 ± 8.16	−2.90 ± 8.32	0.22	0.67
SWMS (Spatial Working memory–Strategy 6–8 boxes)	6.70 ± 3.25	6.85 ± 3.31	0.15 ± 2.18	7.48 ± 2.68	6.76 ± 3.40	−0.71 ± 3.21	0.32	0.67

(Δ): follow-up—baseline values. All data are represented as mean ± standard deviation. * *p*-value for differences between groups estimated by independent samples t-test for change (Δ). *p*(adj): Adjusted *p*-value for false discovery rate using the Benjamini–Hochberg correction method.

**Table 3:** Multivariate regression analysis for the differences in change of cognitive test variables between carnosine and placebo supplementation adjusted for covariates.

Cognitive Tests								
	Models	Change (Δ)						
		β	95% CI	SE	R2	*p* *	P2 (Pooled)	*p*(adj)
Digit Symbol Test Variables								
Score	Model 1	3.99	−2.51, 10.49	3.2	0.38	0.22	0.19	0.34
	Model 2	3.93	−2.82, 10.69	3.32	0.38	0.24	0.21	0.34
	Model 3	3.71	−2.97, 10.40	3.28	0.42	0.27	0.25	0.34
	Model 4	4.03	−2.84, 10.89	3.36	0.42	0.24	0.25	0.34
	Model 5	−0.22	−8.2, −7.77	3.92	0.40	0.96	0.90	0.96
Trail Making Test variables								
Trail Making Test A (s)	Model 1	−2.20	−4.98, 0.58	1.37	0.36	0.12	0.10	0.26
	Model 2	−1.83	−4.73, 1.06	1.43	0.38	0.21	0.17	0.30
	Model 3	−1.83	−4.78, 1.11	1.45	0.38	0.22	0.18	0.30
	Model 4	−2.11	−4.95, 0.73	1.40	0.45	0.14	0.12	0.26
	Model 5	−2.81	−5.75, 0.14	1.45	0.47	0.06	0.05	0.22
Trail Making Test B (s)	Model 1	−17.56	−33.58, −1.54	7.90	0.55	0.03	0.03	0.22
	Model 2	−14.53	−30.71, 1.66	7.97	0.58	0.08	0.07	0.22
	Model 3	−14.81	−31.31, 1.69	8.12	0.58	0.08	0.07	0.22
	Model 4	−15.68	−32.35, 0.99	8.19	0.59	0.06	0.06	0.22
	Model 5	−15.37	−33.10, 2.35	8.71	0.57	0.09	0.09	0.22
B:A (s)	Model 1	−0.59	−1.52, 0.33	0.46	0.37	0.20	0.21	0.30
	Model 2	−0.47	−1.42, 0.48	0.47	0.39	0.32	0.36	0.37
	Model 3	−0.48	−1.45, 0.49	0.48	0.39	0.32	0.36	0.37
	Model 4	−0.46	−1.45, 0.53	0.49	0.39	0.35	0.38	0.37
	Model 5	−0.26	−1.30, 0.79	0.51	0.38	0.62	0.69	0.62
Stroop test variables								
Off Time	Model 1	4.82	−2.27, 11.92	3.45	0.30	0.17	0.09	0.84
	Model 2	4.56	−2.85, 11.98	3.6	0.30	0.22	0.08	0.84
	Model 3	4.31	−3.29, 11.90	3.68	0.31	0.25	0.08	0.84
	Model 4	3.83	−4.11, 11.77	3.84	0.32	0.33	0.15	0.84
	Model 5	0.25	−8.57, 9.07	4.26	0.29	0.95	0.93	0.95
On Time	Model 1	1.77	−12.29, 15.83	6.87	0.03	0.8	0.8	0.89
	Model 2	2.95	−11.27, 17.17	6.94	0.07	0.67	0.67	0.84
	Model 3	2.91	−11.01, 16.83	6.78	0.15	0.67	0.67	0.84
	Model 4	0.82	−12.65, 14.29	6.55	0.25	0.90	0.90	0.93
	Model 5	−4.74	−19.65, 10.18	7.26	0.27	0.52	0.51	0.84
Off Time + On Time	Model 1	11.06	−55.00, 77.12	32.3	0.03	0.38	0.73	0.84
	Model 2	18.58	−47.12, 84.29	32.08	0.10	0.57	0.56	0.84
	Model 3	19.25	−43.94, 82.44	30.8	0.20	0.54	0.53	0.84
	Model 4	8.13	−53.51, 69.76	29.99	0.30	0.79	0.79	0.89
	Model 5	−13.58	−81.90, 54.74	33.24	0.30	0.69	0.68	0.84
On Time–Off Time	Model 1	−2.82	−11.40, 5.76	4.17	0.21	0.51	0.53	0.84
	Model 2	−2.97	−11.81, 5.86	4.29	0.21	0.49	0.54	0.84
	Model 3	−2.83	−11.94, 6.29	4.42	0.21	0.53	0.54	0.84
	Model 4	−4.55	−13.85, 4.75	4.50	0.28	0.32	0.33	0.84
	Model 5	−2.47	−12.35, 7.42	4.78	0.25	0.61	0.52	0.84
Successful times x attempts (Off)	Model 1	22.61	−43.72, 88.95	32.27	0.25	0.49	0.27	0.84
	Model 2	20.19	−48.72, 89.11	33.46	0.26	0.55	0.25	0.84
	Model 3	16.03	−54.28, 86.34	34.07	0.28	0.64	0.25	0.84
	Model 4	16.49	−57.67, 90.66	35.85	0.28	0.65	0.35	0.84
	Model 5	19.37	−60.32, 99.05	38.52	0.28	0.62	0.63	0.84
Successful times x attempts (On)	Model 1	27.76	−47.70, 103.23	36.71	0.16	0.46	0.4	0.84
	Model 2	21.87	−55.82, 99.56	37.72	0.18	0.57	0.4	0.84
	Model 3	14.23	−60.01, 88.46	35.97	0.29	0.7	0.42	0.84
	Model 4	−4.22	−75.03, 66.59	34.23	0.42	0.9	0.66	0.93
	Model 5	−19.89	−96.05, 56.27	36.82	0.43	0.59	0.69	0.84
CANTAB tests								
DMS Percent Correct (All Delays)	Model 1	−2.37	−8.55, 3.80	3.05	0.65	0.44	0.58	0.75
	Model 2	−4.59	−10.76, 1.58	3.05	0.69	0.14	0.31	0.51
	Model 3	−4.96	−10.772, 0.856	2.87	0.74	0.09	0.22	0.50
	Model 4	−5.01	−10.724, 0.698	2.81	0.75	0.08	0.16	0.50
	Model 5	−6.86	−12.60, −1.11	2.83	0.77	0.02	0.03	0.31
DMS percent correct (Simultaneous)	Model 1	−2.75	−7.63, 2.13	2.41	0.66	0.26	0.34	0.58
	Model 2	−4.39	−9.36, 0.59	2.46	0.69	0.11	0.21	0.50
	Model 3	−4.63	−9.40, 0.15	2.36	0.73	0.08	0.15	0.50
	Model 4	−4.67	−9.29, −0.052	2.28	0.75	0.06	0.10	0.50
	Model 5	−0.97	−6.57, 4.66	2.77	0.57	0.73	0.67	0.92
DMS Percent Correct (0 Second Delay)	Model 1	4.933	−5.30, 15.17	5.05	0.66	0.34	0.30	0.68
	Model 2	2.868	−7.82, 13.56	5.28	0.67	0.59	0.48	0.83
	Model 3	2.134	−8.21, 12.48	5.10	0.70	0.68	0.57	0.90
	Model 4	2.097	−8.25, 12.44	5.10	0.71	0.68	0.62	0.90
	Model 5	4.09	6.4, 14.57	5.16	0.72	0.43	0.40	0.74
DMS Percent Correct (4 Second Delay)	Model 1	−6.04	−16.41, 4.34	5.13	0.51	0.25	0.39	0.58
	Model 2	−8.81	−19.65, 2.04	5.35	0.54	0.11	0.27	0.50
	Model 3	−9.14	−20.09, 1.80	5.40	0.55	0.1	0.24	0.50
	Model 4	−9.03	−20.13, 2.07	5.47	0.55	0.11	0.25	0.50
	Model 5	−13.03	−24.37,−2.24	5.45	0.58	0.02	0.03	0.31
DMS Percent Correct (12 Second Delay)	Model 1	−5.98	−16.53, 4.57	5.21	0.60	0.26	0.27	0.58
	Model 2	−6.94	−17.89, 4.01	5.40	0.61	0.21	0.23	0.54
	Model 3	−7.74	−18.51, 3.04	5.31	0.63	0.15	0.16	0.51
	Model 4	−8.28	−18.61, 2.06	5.09	0.67	0.11	0.09	0.50
	Model 5	−9.92	−20.19, 0.35	5.06	0.68	0.06	0.05	0.50
DMS median correct latency	Model 1	−140.46	−886.88, 605.95	368.71	0.21	0.71	0.72	0.92
	Model 2	−120.18	−908.07, 667.70	388.85	0.21	0.76	0.77	0.92
	Model 3	−115.96	−916.68, 684.76	394.81	0.21	0.77	0.78	0.92
	Model 4	−126.76	−904.05, 650.54	382.88	0.28	0.74	0.67	0.92
	Model 5	−50.32	−844.91, 744.26	391.4	0.27	0.9	0.87	0.97
DMS Probability of error given error	Model 1	0.02	−0.09, 0.13	0.05	0.62	0.70	0.79	0.91
	Model 2	0	−0.12, 0.12	0.06	0.62	0.99	0.60	0.99
	Model 3	−0.01	−0.13, 0.12	0.06	0.63	0.9	0.56	0.97
	Model 4	0	−0.13, 0.12	0.06	0.65	0.94	0.58	0.97
	Model 5	−0.04	−0.15, 0.08	0.06	0.65	0.50	0.52	0.79
PALFAMS (Paired Associates Learning First)	Model 1	−2.19	−4.60, 0.23	1.19	0.3	0.07	0.06	0.50
	Model 2	−2.93	−5.49, −0.37	1.26	0.34	0.03	0.03	0.36
	Model 3	−2.95	−5.55, −0.34	1.28	0.34	0.03	0.03	0.36
	Model 4	−2.83	−5.46, −0.21	1.29	0.36	0.04	0.04	0.41
	Model 5	−3.22	−5.84,−0.61	1.28	0.36	0.02	0.006	0.31
PALMETS (Paired Associates Learning Mean Errors to Success)	Model 1	1.491	0.81, 2.17	0.34	0.65	0.001	0.001	0.15
	Model 2	1.72	1.01, 2.43	0.35	0.68	0.001	0.001	0.41
	Model 3	1.76	1.06, 2.47	0.35	0.7	0.001	0.001	0.31
	Model 4	1.70	1.00, 2.39	0.34	0.72	0.001	0.001	0.31
	Model 5	1.42	0.63, 2.22	0.39	0.65	0.001	0.001	0.50
PALTA (Paired Associates Learning Total Attempts)	Model 1	1.10	0.08, 2.13	0.50	0.41	0.04	0.03	0.73
	Model 2	1.47	0.42, 2.52	0.52	0.47	0.01	0.01	0.73
	Model 3	1.44	0.39, 2.48	0.51	0.49	0.01	0.01	0.74
	Model 4	1.30	0.31, 2.29	0.49	0.57	0.01	0.02	0.54
	Model 5	1.40	0.38, 2.41	0.50	0.57	0.008	0.01	0.97
PALTA 4 (Paired Associates Learning Total Attempts 4 patterns)	Model 1	0.46	−0.04, 0.96	0.25	0.24	0.07	0.06	0.90
	Model 2	0.25	−0.35, 0.85	0.29	0.29	0.40	0.19	0.90
	Model 3	0.25	−0.35, 0.85	0.30	0.30	0.40	0.19	0.91
	Model 4	0.24	−0.37, 0.85	0.30	0.31	0.42	0.21	0.58
	Model 5	0.36	−0.19, 0.91	0.27	0.30	0.20	0.09	0.36
PALTEA (Paired Associates Learning Total errors (adjusted))	Model 1	−0.33	−7.32, 6.66	3.45	0.14	0.92	0.89	0.31
	Model 2	1.517	−5.94, 8.97	3.67	0.19	0.68	0.88	0.31
	Model 3	1.53	−6.044, 9.10	3.72	0.19	0.68	0.87	0.31
	Model 4	1.459	−6.252, 9.17	3.79	0.19	0.70	0.86	0.31
	Model 5	4.42	−3.28, 12.12	3.78	0.22	0.25	0.23	0.67
PRMPCD (Pattern Recognition Memory Percent Correct Delayed)	Model 1	−9.21	−17.38, −1.05	4.02	0.37	0.03	0.05	0.54
	Model 2	−10.9	−19.47, −2.32	4.22	0.4	0.01	0.04	0.54
	Model 3	−10.72	−19.36, −2.08	4.24	0.41	0.02	0.05	0.54
	Model 4	−10.73	−19.52, −1.93	4.31	0.41	0.02	0.05	0.50
	Model 5	−11.27	−20.23,−2.31	4.39	0.42	0.02	0.04	0.82
PRMMDCLD (Pattern Recognition Memory Median correct latency)–Delayed	Model 1	188.12	−194.09, 570.33	188.07	0.42	0.32	0.26	0.92
	Model 2	263.75	−144.49, 671.99	200.66	0.44	0.20	0.15	0.92
	Model 3	261.01	−143.24, 665.25	198.46	0.47	0.20	0.17	0.94
	Model 4	257.24	−156.45, 670.93	202.84	0.47	0.21	0.20	0.92
	Model 5	358.25	−39.9, −756.41	195.22	0.49	0.08	0.09	0.62
PRMMDCLI (Pattern Recognition Memory median correct latency)–Immediate	Model 1	40.2	−102.57, 182.97	70.46	0.32	0.57	0.43	0.58
	Model 2	20.18	−131.65, 172.02	74.87	0.33	0.79	0.53	0.58
	Model 3	22.45	−131.53, 176.43	75.85	0.33	0.77	0.51	0.58
	Model 4	15.91	−139.09, 170.92	76.27	0.35	0.84	0.61	0.53
	Model 5	24.53	−129.81, 178.87	75.95	0.35	0.75	0.64	0.51
PRMPCI (Pattern Recognition Memory Percent Correct)–Immediate	Model 1	−3.26	−9.28, 2.75	2.97	0.29	0.28	0.31	0.50
	Model 2	−3.62	−10.01, 2.76	3.15	0.29	0.26	0.32	0.50
	Model 3	−3.68	−10.17, 2.81	3.20	0.29	0.26	0.31	0.51
	Model 4	−3.77	−10.36, 2.81	3.24	0.29	0.25	0.29	0.56
	Model 5	−4.45	−10.98, 2.09	3.21	0.31	0.18	0.18	0.54
RTIFMMT (Reaction Time Index–mean five choice movement time)	Model 1	29.6	−10.70, 69.89	19.92	0.22	0.15	0.14	0.53
	Model 2	33.62	−8.32, 75.56	20.72	0.23	0.11	0.100	0.51
	Model 3	35.96	−4.07, 75.98	19.75	0.32	0.08	0.07	0.54
	Model 4	31.13	−8.99, 71.24	19.78	0.36	0.12	0.12	0.62
	Model 5	25.16	−15.72, 66.04	20.16	0.34	0.22	0.21	0.97
RTIFMDMT (RTI Median Five-Choice Movement Time)	Model 1	25.81	−15.07, 66.69	20.21	0.20	0.21	0.20	0.97
	Model 2	29.31	−13.24, 71.87	21.02	0.21	0.17	0.16	0.93
	Model 3	31.55	−9.22, 72.32	20.12	0.30	0.13	0.12	0.92
	Model 4	26.72	−14.20, 67.64	20.18	0.33	0.19	0.19	0.93
	Model 5	21.88	−19.71, 93.3	20.51	0.32	0.29	0.29	0.80
RTIFMTSD (Reaction Time Index -five choice movement time (SD))	Model 1	−0.45	−10.95, 10.06	5.19	0.3	0.93	0.93	0.83
	Model 2	0.50	−10.44, 11.43	5.40	0.31	0.93	0.93	0.80
	Model 3	1.29	−9.04, 11.63	5.10	0.40	0.80	0.80	0.81
	Model 4	1.43	−9.06, 11.92	5.17	0.40	0.78	0.78	0.92
	Model 5	1.27	−9.55, 12.1	5.34	0.40	0.81	0.81	0.97
RTISES (Reaction time Index–Simple error Score)	Model 1	0.29	−0.64, 1.22	0.46	0.09	0.53	0.52	0.92
	Model 2	0.26	−0.71, 1.23	0.48	0.09	0.59	0.58	0.92
	Model 3	0.30	−0.67, 1.27	0.48	0.12	0.54	0.53	0.97
	Model 4	0.29	−0.70, 1.28	0.49	0.12	0.55	0.55	0.79
	Model 5	−0.35	−2.88, 2.18	1.25	0.14	0.78	0.78	0.53
RTIMSMT (Reaction Time Index–Mean simple movement time)	Model 1	−1.54	−34.35, 31.28	16.22	0.30	0.92	0.92	0.53
	Model 2	4.38	−29.24, 38.01	16.61	0.33	0.79	0.79	0.51
	Model 3	5.39	−27.92, 38.69	16.44	0.36	0.74	0.74	0.53
	Model 4	2.12	−31.15, 35.39	16.4	0.40	0.90	0.90	0.85
	Model 5	−10.97	−44.42, 22.49	16.5	0.40	0.50	0.51	0.53
RTIMSRT (Reaction Time Index–Mean simple reaction time)	Model 1	−12.89	−32.14, 6.36	9.52	0.19	0.18	0.18	0.51
	Model 2	−13.81	−33.88, 6.26	9.91	0.19	0.17	0.16	0.51
	Model 3	−14.44	−34.56, 5.67	9.93	0.22	0.15	0.15	0.51
	Model 4	−13.66	−33.88, 6.55	9.97	0.24	0.18	0.17	0.50
	Model 5	−19.53	−39.99, 0.93	10.09	0.27	0.61	0.05	0.92
RTISMDRT (RTI Simple Median Reaction Time)	Model 1	−11.25	−27.94, 5.44	8.25	0.22	0.18	0.17	0.98
	Model 2	−12.52	−29.87, 4.83	8.57	0.23	0.15	0.14	0.97
	Model 3	−13.23	−30.47, 4.01	8.51	0.26	0.13	0.12	0.97
	Model 4	−12.34	−29.49, 4.82	8.46	0.29	0.15	0.14	0.74
	Model 5	−15.31	−32.87, 2.25	8.66	0.31	0.09	0.08	0.94
RTISMDMT (RTI Simple Median Movement Time)	Model 1	−4.54	−35.65, 26.57	15.38	0.31	0.77	0.77	0.94
	Model 2	0.62	−31.41, 32.65	15.82	0.34	0.97	0.97	0.94
	Model 3	1.55	−30.21, 33.32	15.68	0.37	0.92	0.92	0.94
	Model 4	−1.15	−33.04, 30.75	15.73	0.39	0.94	0.94	0.87
	Model 5	−12.68	−44.72, 19.37	15.8	0.40	0.43	0.42	0.75
RVPA (Rapid Visual Processing A prime)	Model 1	0	−0.03, 0.03	0.01	0.20	0.84	0.75	0.77
	Model 2	0	−0.03, 0.03	0.02	0.20	0.84	0.69	0.73
	Model 3	0	−0.04, 0.03	0.02	0.20	0.85	0.7	0.73
	Model 4	0	−0.04, 0.03	0.02	0.21	0.85	0.69	0.74
	Model 5	0.01	−0.02, 0.04	0.02	0.22	0.63	0.89	0.92
RVPMDL (Rapid Visual Processing–median response latency)	Model 1	12.81	−22.02, 47.65	17.19	0.35	0.46	0.30	0.97
	Model 2	12.92	−24.16, 50.01	18.29	0.35	0.48	0.28	0.97
	Model 3	15.91	−20.31, 52.13	17.84	0.41	0.38	0.20	0.98
	Model 4	15.58	−0.59, −0.15	17.95	0.42	0.39	0.25	0.68
	Model 5	15.03	−22.29, 52.36	18.36	0.42	0.42	0.33	0.75
RVPPFA (RVP Probability of False Alarm)	Model 1	0.002	−0.02, 0.02	0.01	0.90	0.79	0.78	0.80
	Model 2	0.001	−0.02, 0.02	0.01	0.90	0.95	0.92	0.80
	Model 3	0.001	−0.02, 0.02	0.01	0.90	0.95	0.92	0.77
	Model 4	0	−0.02, 0.02	0.01	0.91	0.97	0.90	0.80
	Model 5	−0.001	−0.03, 0.01	0.01	0.91	0.34	0.35	0.54
SWMS (Spatial Working memory–Strategy 6–8 boxes)	Model 1	−0.64	−2.33, 1.06	0.84	0.12	0.45	0.43	0.58
	Model 2	−0.54	−2.31, 1.24	0.88	0.13	0.54	0.51	0.53
	Model 3	−0.56	−2.36, 1.24	0.89	0.13	0.53	0.50	0.51
	Model 4	−0.61	−2.34, 1.11	0.85	0.22	0.48	0.39	0.62
	Model 5	0.57	−1.19, 2.33	0.87	0.22	0.52	0.55	0.67
SWMBE (Spatial Working Memory–between errors)	Model 1	−3.2	−8.28, 1.88	2.51	0.25	0.21	0.20	0.75
	Model 2	−3.2	−8.56, 2.17	2.65	0.25	0.24	0.22	0.73
	Model 3	−3.56	−8.62, 1.50	2.50	0.35	0.16	0.14	0.74
	Model 4	−3.68	−8.67, 1.32	2.46	0.39	0.14	0.11	0.84
	Model 5	−2.73	−7.9, 2.44	2.55	0.37	0.29	0.26	0.50
SWMBE4 (SWM Between Errors 4 Boxes)	Model 1	−0.34	−1.01, 0.34	0.33	0.33	0.32	0.37	0.51
	Model 2	−0.27	−0.96, −0.34	0.35	0.34	0.45	0.51	0.50
	Model 3	−0.30	−0.99, 0.40	0.34	0.37	0.40	0.45	0.50
	Model 4	−0.29	−1.00, 0.42	0.35	0.38	0.41	0.47	0.79
	Model 5	−0.19	−0.91, 0.53	0.36	0.37	0.60	0.63	0.82
SWMBE6 (Spatial Working Memory–between errors 6 boxes)	Model 1	−1.68	−3.40, 0.05	0.85	0.47	0.06	0.04	0.75
	Model 2	−1.38	−3.17, 0.40	0.88	0.49	0.12	0.08	0.70
	Model 3	−1.46	−3.21, 0.28	0.86	0.53	0.10	0.06	0.68
	Model 4	−1.49	−3.24, 0.26	0.86	0.54	0.09	0.06	0.70
	Model 5	−0.60	−2.46, 1.25	0.91	0.50	0.51	0.46	0.75
SWMBE8 (Spatial Working Memory–between errors 8 boxes)	Model 1	−1.1	−5.03, 2.83	1.94	0.27	0.57	0.56	0.51
	Model 2	−1.57	−5.69, 2.55	2.03	0.28	0.45	0.45	0.50
	Model 3	−1.81	−5.77, 2.15	1.95	0.36	0.36	0.35	0.50
	Model 4	−1.90	−5.81, 2.01	1.92	0.39	0.33	0.29	0.31
	Model 5	−1.83	−5.82, 2.17	1.97	0.39	0.36	0.33	0.58

Model 1: Adjusted for baseline value. Model 2: Adjusted for baseline value, diabetic status. Model 3: Adjusted for baseline value, diabetic status and level of education. Model 4: Adjusted for baseline value, diabetic status, level of education and age. Model 5: Interaction term (Group × Sex) and adjusted for baseline value, diabetic status, level of education and age. * *p*-values are estimated by Analysis of Covariance (ANCOVA). P2(Pooled): Pooled *p*-values estimated by ANCOVA after 5 imputations using predictive mean matching to replace missing data. *p*(adj): Adjusted *p*-value for false discovery rate using the Benjamini–Hochberg correction method. (β) Unstandardized beta-coefficient. (CI) confidence interval. (SE) standard error, and. (R2) R-square value.

## Data Availability

The data presented in this study can be made available upon reasonable request to the corresponding author. The data are not publicly available due to participant confidentiality agreements, per the conditions of the ethics approval for the trial.

## Supplementary Materials

The following supporting information can be downloaded at https://www.mdpi.com/article/10.3390/ph18050630/s1.
