# Supplementary material for: Effects of Carnosine Supplementation on Cognitive Outcomes in Prediabetes and Well-Controlled Type 2 Diabetes: A Randomised Placebo-Controlled Clinical Trial
Source: Pharmaceuticals (Basel). 2025 Apr 26;18(5):630. doi: 10.3390/ph18050630 (PMC12114902; doi:10.3390/ph18050630)
Supplement: Supplementary file 1 [file pharmaceuticals-18-00630-s001.zip › Supplementary Sections.pdf]

Effects of carnosine supplementation on cognitive outcomes in prediabetes and well-controlled type 2 diabetes: a randomised placebo-controlled clinical trial

Supplementary Sections

Supplementary S1: Methods

(1) Digit-Symbol substitution test: a measure of cognitive flexibility, attention span and executive function

First, each participant completed seven practice boxes, after which a two-minute timer was started. Participants were encouraged to focus and answer as many numbered boxes as possible, and to stop answering when the time was complete, with 93 as the maximum attainable score. The answers were checked by the tester; correct reproduction and placement of the symbol with the digit was scored as 1 point out of a maximum of 93.

(2) STROOP test: a measure of cognitive flexibility and processing speed

Participants completed in turn 14 runs where the test alternated between the Stroop "off" state – a series of "####" symbols appearing and disappearing in three different colours (red, blue, green) at different locations on the iPad screen and the Stroop "on" state – the words "red", "green" and "blue" appearing in colours incongruent with the descriptor. Participants were required to press the appropriate response buttons labelled with the colours at the bottom of the screen. Response button order and colour were continuously changed, and participants were required to respond based on the colour and not the word. Any mistakes made by the participant restarted the specific run. The application recorded the time spent on each run of the STROOP test and also provided a combined estimate of time taken to complete each state of the STROOP test.

(3) Trail Making Test: a measure of executive functioning

In part A of this test, participants used the index finger of their dominant hand to trace a line from numbers 1 to 15 displayed in circles in random order on the iPad screen. Participants were instructed to connect the circles in order without lifting their finger once the test clock was initiated. In part B of the test, participants performed the same task, but with numbers and letters. Participants were required to trace from a number to a corresponding letter, e.g.: "1-A, 2-B, 3-C, 4-D etc." under timed settings. If

the participants made an error, they were immediately corrected by the tester before they moved to the next target, and only one run was administered. The application measured time (seconds) spent to complete each part of the test.

(4) CANTAB: a measure of cognitive flexibility, attention span, executive function, processing speed and visuospatial processing.

- *Delayed match to sample:* Participants are required to accurately match a visually complex pattern displayed at the centre of the screen briefly and then obscured. After a small delay, three patterns which are visually similar and one which is identical to the test pattern, are displayed on the screen. To succeed, the participant must only select the visually identical pattern to the test pattern displayed at the start of the test. Different levels of delay (0, 4 or 12 seconds) are used between displaying the test and response patterns.[43]

- *Paired associates learning:* Participants are presented with 6 boxes, each with a unique pattern that is initially obscured and then revealed one at a time in random order and concealed again. Participants are then shown a pattern in the middle of the screen and are required to select the box in which they had observed this pattern earlier. If a mistake is made, all patterns are reshown, and the test restarted until the maximum error threshold is reached. If the participant answers correctly, they are progressed to the next phase. The easiest stage requires one correct answer, and the hardest stage requires eight.[43]

- *Pattern recognition memory:* In the second phase, participants are shown two patterns: one seen before and the other novel. The participant then selects the pattern seen before. The test then reverses the order of patterns, and the participant repeats the same task. This is continued until the participant has provided sufficient attempts. In the final phase, the test introduces a six-minute delay. On recommencement, the participant is asked to select from a final choice of two patterns to collect the delayed phase response.

- *Reaction time index*: Five empty circles arranged in an arc are displayed at the top of screen. Participants are asked to enter their start position by pressing a button at the bottom of the screen with the index finger of their dominant hand and holding their finger in position. This action initiates the appearance of a green dot in one of the five circles in a random order. The participants are required to let go of the button and touch the green dot, then return their index finger to the start position. The test prompts the participants if they are too fast, too slow or if they miss the target. The application then measures reaction time in seconds.

- *Rapid visual processing:* Participants are instructed to press a button located at the bottom of the screen only after they have seen the last number of the sequence: '3-5-7'.Initially the test indicates these digits in red and all other numbers in white. After this phase, the red colouring disappears. In the next phase, two more sequences are added: "4-6-8" and "2-4-6".[43] The application then determines the participant's sensitivity in detecting the target sequences and also specificity by calculating: False Alarms ÷ (False Alarms + Correct Rejections).

- *Spatial working memory:* Participants are presented with eight yellow onscreen boxes and told that only one box has a blue token within. Participants search the boxes for the token and, when found, are instructed to store the token by tapping/dragging to an empty box located at the bottom of the screen. Participants are also reminded that boxes in which tokens are found, will not contain another token in that run. Difficulty is moderated by utilising smaller or greater number of tokens to be found.[43] Two scores are calculated: 1) the number of times the participant incorrectly revisits a box, calculated across four, sex and eight trials 2) the number of different strategies used by the participant when starting a new search for a token from the same starting box, assessed across 6 to 8 boxes.

Supplementary S2: Results

Differences in the digit symbol substitution test (DSST) between carnosine and placebo:

Scores of the DSST in each group are presented in (**Table 2)**. There were no statistically significant differences between the groups in change values for DSST scores (p=0.46). The multivariable regression analysis **(Table 3)** showed no effects of treatment allocation adjusted for baseline DSST score (p=0.34), diabetic status (p=0.34), level of education (p=0.34) or age (p=034) or interaction effects with sex (P=0.96)

Differences in STROOP between carnosine and placebo:

Results for Stroop for each group are presented in **Table 2 & 3/Table S3.1 & 3.2**. There were no significant differences in change values for "Off Time" (p=0.75) between groups. There were also no differences in "Off Time" change values after adjustment for baseline STROOP score values and diabetic status, level of education or age (all p>0.67). Similarly, in the measure "Off time + On Time", there were no effects of treatment allocation in the multivariable linear regression models when adjusting for baseline values, diabetic status, level of education or age (all p>0.8) (**Table 3/Table S3.1 &3.2).** In the interference state, "Ontime – Offtime", there were no significant differences in change values between groups (p=0.68), including after adjustment for the same covariates (all p>0.84). There were no differences in the subgroup analysis of prediabetes compared to diabetes **(Table S3.3)** or interaction effects with sex (p=0.84).

Differences in the trail-making test (TMT-A & B) between carnosine and placebo:

The results for TMT-A and TMT-B are presented in **Table 2 & 3**. There were no significant differences in change values for either part of the Trail Making between the carnosine and the placebo groups (TMT-A, p=0.76; TMT-B, p=0.42; B: A, p=0.66) (**Table 2**). In the multivariable regression analysis (**Table 3**), there were no differences between carnosine and placebo in TMT-A, TMT-B or B: A after adjusting for baseline values, diabetic status, level of education or age (all p>0.22) or interaction effects with sex (p>0.22). Similarly, there were no significant differences in a subgroup analysis of prediabetes versus diabetes **(Table S4)**.

Differences in CANTAB between carnosine and placebo

Results from the CANTAB tests are presented in **Table 2 & 3/Table S5.1**. For the *Delayed Match to Sample (DMS)* test, the key variables analysed were DMS % correct (All Delays), DMS % correct simultaneous (DMSPC), DMS median latency (DMSMDLAD) and DMS probability of error given error (DMSPEGE). There were no differences in change values between carnosine and placebo groups in any of these DMS t-tests. The multivariable

linear regression models (**Table 3**) for DMS showed no effect of treatment allocation after adjusting for baseline values, diabetic status, level of education or age or interaction effects with sex (all p>0.31). Similarly, in a sub-group analysis of prediabetes versus diabetes, there were no differences between carnosine and placebo groups **(Table S5.2.1, 5.2.2, 5.2.3, 5.2.4)**.

In the domain of *Paired Associates Learning (PAL)*, 4 key variables were analysed including PAL first attempt memory score (PALFAMS) that showed no significant difference between carnosine and placebo groups (p=0.54), and no significant effect of treatment allocation on multivariable regression analysis (all p≥0.31) (**Table 3**) or interaction effects with sex (p=0.31). There were no significant differences between pre-diabetics and diabetics in the subgroup analysis for PALFAMS **(Table S5.2.1, 5.2.2, 5.2.3, 5.2.4)**. For the measure PAL total errors adjusted (PALTEA); there were no differences in change values between groups (p=0.86). For the variable PAL mean errors to success (PALMETS), there no were significant differences in change values between carnosine and placebo groups (p=0.05), including after adjustment for baseline values, diabetic status, level of education and age (all p>0.31) (**Table 3**). For the measure PAL total attempts (PALTA) or the more sensitive PAL Total Attempts 4 patterns (PALTA4), there were no significant differences (p=0.46 and p=0.59, respectively) (**Table 2**) between carnosine and placebo groups, including in multivariable regression adjusted for the same covariates or sex interactions (all p≥0.54 and p≥0.36 for PALTA and PALTA4, respectively). In a sub-group analysis comparing prediabetes and diabetes, after applying the Benjamini-Hochberg (BH) correction, the prediabetes group was significant after Benjamini-Hochberg correction (p=0.03), suggesting a subgroup effect. However, significance was lost in multivariable regression, likely due to the small sample size, warranting validation in a larger cohort of participants with prediabetes (**Table S5.2.1, S5.2.2**). There were no other subgroup differences or interaction effects by sex (p>0.35) (**Table 3**).

In the domain of *Pattern Recognition Memory (PRM)*, 4 key variables were analysed including 'PRM percent correct latency – delayed'; 'PRM percent correct latency – immediate'; 'PRM median correct latency – delayed'; and 'PRM median correct latency – immediate'. There were no significant differences between carnosine and placebo groups in any measure of PRM (all p>0.60). The multivariable regression analysis of the PRM measures showed no effect of treatment allocation when adjusting for baseline values, diabetic status, level of education or age (all p>0.5) (**Table 3**). There were no significant findings in the subgroup analysis (**Table S5.2.1, 5.2.2, 5.2.3, 5.2.4**) or interaction effects with sex (all p>0.51) (**Table 3**).

The *Reaction Time index (RTI)* evaluated twenty measures as presented in **Table 2 & 3**. There were no significant differences in change in any RTI measure between carnosine and placebo groups (all p>0.46), including after adjustment for covariates (all p> 0.50) and in the subgroup analysis or interaction effects with sex (all p>0.5).

For the *Rapid Visual Processing (RVP)* domain, nine variables were assessed in the analysis including key variables: RVP-A prime (RVPA) and RVP-median response latency (RVPMDL) and RVP probability of false alarm (RVPPFA) (**Table 2 & 3**). There were no significant differences in changes between carnosine and placebo groups for any measure of RVP (**Table 2, 3**). Further analysis using multivariable regression or subgroup analysis and interaction effects with sex (all p>0.5) found no significant effects of treatment allocation.

The analysis for the *Spatial Working Memory (SWM)* domain, included 2 key variables: SWM-between errors (SWMBE), SWM-strategy (SWMS). There were no significant differences in the changes of these variables between carnosine and placebo groups, including in multivariable analysis (all p>0.30) (**Table 3**) and subgroup analysis (**Table S5.2.1, 5.2.2, 5.2.3, 5.2.4**) or interaction effects with sex (all p>0.5).
