# Supplementary material for: Effects of Carnosine Supplementation on Cognitive Outcomes in Prediabetes and Well-Controlled Type 2 Diabetes: A Randomised Placebo-Controlled Clinical Trial
Source: Pharmaceuticals (Basel). 2025 Apr 26;18(5):630. doi: 10.3390/ph18050630 (PMC12114902; doi:10.3390/ph18050630)
Supplement: Supplementary file 1 [file pharmaceuticals-18-00630-s001.zip › Supplementary tables.pdf]

Supplementary Section

Effects of Carnosine on Cognitive Outcomes in Prediabetes and Well-Controlled Type 2 Diabetes: Results from a Randomised Placebo-Controlled Clinical Trial

Table S1. Sample characteristics at baseline

**Stroop**		
Total # of runs stroop off	5.688 ± 0.873	5.533 ± 0.64
Total # of runs stroop on	5.813 ± 1.424	5.667 ± 0.816
**Delayed match to sample (DMS)**		
DMS Mean choices to correct	1.13 ± 0.08	1.15 ± 0.14
DMS Correct latency	1226.99 ± 534.42	1009.97 ± 598.18
DMS Correct latency (0s delay)	1225.72 ± 890.07	1236.78 ± 1026.88
DMS Correct latency (12 s delay)	857.11 ± 835.86	1203.15 ± 620.46
DMS Correct latency (4 s delay)	1352.25 ± 803.33	1169.76 ± 781.03
DMS Correct latency (all delays)	1361.34 ± 617.77	1123.98 ± 623.24
DMS Correct latency (simultaneous)	747.20 ± 541.31	812.20 ± 394.15
DMS Median correct latency	3473.00 (1095.63)	2924.00 (999.50)
DMS Median correct latency (0 s delay)	2926.25 (1047.00)	2706.25 (1268.13)
DMS Median correct latency (12 s delay)	3535.75 (1155.75)	3348.00 (1319.50)
DMS Median correct latency (4 s delay)	4264.50 (1845.00)	3093.00 (2013.00)
DMS Median correct latency (all delays)	3740.50 (1254.63)	2896.00 (1096.00)
DMS Median correct latency (simultaneous)	3011.00 (1325.25)	2885.00 (1099.50)
DMS Mean correct latency	3586.84 ± 833.47	3606.9 ± 1542.73
DMS Mean correct latency (0 s delay)	3417.07 ± 1021.86	3441.5 ± 1446.84
DMS Mean correct latency (12 s delay)	3905.54 ± 1461.52	3844.02 ± 1894.79
DMS Mean correct latency (4 s delay)	4138.2 ± 1245.67	3881.77 ± 1939.08
DMS Mean correct latency (all delays)	3825.89 ± 949.7	3784.71 ± 1704.98
DMS Mean correct latency (simultaneous)	2987.015 ± 754.12	3142.698 ± 1339.82
DMS Percent correct (0 s delay)	100 (20)	100 (20)
DMS Percent correct (12 s delay)	80 (40)	80 (20)
DMS Percent correct (4 s delay)	90 (20)	80 (40)
DMS Percent correct (all delays)	87.00 (13.00)	87.00 (13.00)
DMS Percent correct (simultaneous)	100 (0)	100 (0)
DMS Total correct (0 s delay)	5.00 (1.00)	5.00 (1.00)
DMS Total correct (12 s delay)	4.00 (2.00)	4.00 (1.00)
DMS Total correct (4 s delay)	4.50 (1.00)	4.00 (2.00)
DMS Total correct (all delays)	13.00 (2.00)	13.00 (2.00)
DMS Total Errors	2.00 (2.00)	2.00 (2.00)
DMS Total Errors (All Delays)	2.00 (2.00)	2.00 (2.00)
DMS Error (incorrect colour)	0 (1.00)	1.00 (1.00)
DMS Error (all delays, incorrect colour)	0 (1.00)	1.00 (1.00)
DMS Error (Distractor)	0 (0)	0 (0)
DMS Error (all delays, distractor)	0 (0)	0 (0)
DMS Error (incorrect pattern)	2.00 (1.00)	2.00 (2.00)
DMS Error (all delays, incorrect pattern)	2.00 (1.00)	1.00 (2.00)
**Motor Screening Task (MOT)**		
MOT Median latency	109.77 (137.55)	138.55 (139.38)
**Paired Associates Learning (PAL)**		
PAL Total attempts 2 patterns	1.00 (0)	1.00 (0)
PAL Total attempts 4 patterns	2.00 (1.00)	1.00 (0)

Effects of Carnosine on Cognitive Outcomes in Prediabetes and Well-Controlled Type 2 Diabetes: Results from a Randomised Placebo-Controlled Clinical Trial

PAL Total attempts 6 patterns	3.00 (1.50)	2.00 (2.00)
PAL Total attempts 8 patterns	4.00 (2.00)	3.50 (2.00)
PAL Total errors 2 patterns	0 (0)	0 (0)
PAL Total errors 4 patterns	1.00 (2.00)	0 (0)
PAL Total errors 6 patterns	4.00 (4.00)	2.50 (5.50)
PAL Total errors 8 patterns	6.00 (7.50)	8.50 (13.00)
PAL Total errors 2 shapes adjusted	0 (0)	0 (0)
PAL Total errors 4 shapes adjusted	1.00 (2.00)	0 (0)
PAL Total errors 6 shapes adjusted	4.00 (4.00)	2.50 (5.50)
PAL Total errors 8 shapes adjusted	7.00 (10)	11.00 (12.50)
**Reaction Time Index (RTI)**		
RTI Five-choice error score (inaccurate)	0 (0)	0 (0)
RTI Five choice error score (no response)	0 (0)	0 (0)
RTI Five choice error score (premature)	0 (0)	0 (0)
RTI Median five choice movement time	292.00 (130.50)	244.50 (59.00)
RTI Median five choice reaction time	415.50 (59.00)	396.00 (70.50)
RTI Mean five choice movement time	292.091 ± 82.544	255.142 ± 86.815
RTI Mean five choice reaction time	411.603 ± 46.920	411.581 ± 44.116
RTI Five-choice movement time: Standard Deviation	40.310 ± 17.223	37.491 ± 14.087
RTI Five-choice reaction time	47.04 (22.90)	46.05 (23.00)
RTI Simple error score (inaccurate)	0 (1.00)	0 (1.00)
RTI Simple error score (no response)	0 (0)	0 (0)
RTI Simple error score (premature)	0 (1.00)	0 (1.00)
RTI Median simple reaction time	349.00 (58.00)	333.00 (67.00)
RTI Mean simple movement time	232.699 ± 75.089	207.118 ± 79.628
RTI Mean simple reaction time	353.960 ± 38.800	350.280 ± 42.604
RTI Median simple movement time	34.32 (20.42)	25.05 (9.97)
**Pattern Recognition Memory**		
PRM average correct latency - Delayed	2144.31 (649.71)	1955.80 (689.36)
PRM average correct latency - Immediate	1708.91 (496.19)	1665.50 (493.60)
Rapid visual processing (RVP)		
RVP Median response latency	457.25 (98.75)	460 (68.00)
RVP Mean response latency	550.6 ± 113.53	531.99 ± 71.51
RVP Probability of false alarm	0.01 (0.02)	0 (0.01)
RVP Probability of hit	0.57 (0.19)	0.67 (0.24)
**Spatial working memory (SWM)**		
SWM Double errors	0 (1.00)	0 (1.00)
SWM Problem Reached	5.00 (0)	5.00 (0)
SWM Total errors	10.50 (19.75)	12.00 (10)
SWM Between errors 4 boxes	0 (2.00)	0 (0)
SWM Between errors 6 boxes	0.50 (6.25)	5.00 (6.00)
SWM Between errors 8 boxes	9.50 (13.25)	8.00 (10)
SWM Double errors 4 boxes	0 (0)	0 (0)
SWM Double errors 6 boxes	0 (0)	0 (0)
SWM Double errors 8 boxes	0 (0.25)	0 (0)
SWM Strategy (6- 8 boxes)	3.00 (3.00)	4.00 (1.00)
SWM Total errors 4 boxes	0 (2.00)	0 (0)
SWM Total errors 6 boxes	0.50 (6.25)	5.00 (6.00)
SWM Total errors 8 boxes	10 (14.00)	8.00 (10)

SWM Within errors	0 (1.00)	0 (1.00)
SWM Within errors 4 boxes	0 (0)	0 (0)
SWM Within errors 6 boxes	0 (0)	0 (0)
SWM Within errors 8 boxes	0 (1.00)	0 (1.00)

Table S2: Digit Symbol Tests subgroup analysis

Table S2.1 Prediabetes: Digit Symbol test variables between carnosine supplementation and placebo.

Stroop test variables	Placebo			Carnosine			*P**	*P2 (Pooled)*	*P(adj)*
	**Baseline**	**Follow-Up**	**Δ**	**Baseline**	**Follow-Up**	**Δ**			
**Score**	70.54±11.48	75.33±12.94	3.92±11.18	63.78±20.04	74.78±11.58	11±14.05	0.21	0.18	0.64

(Δ): follow-up - baseline values.
All data are represented as Mean ± Standard Deviation.
* p-value for differences between groups estimated by independent samples t-test for change (Δ).
P2(Pooled): Pooled p-values estimated by ANCOVA after 5 imputations using predictive mean matching to replace missing data.
P(adj): Adjusted p-value for false discovery rate using Benjamini-Hochberg correction method.

Table S2.2 Diabetes: Digit Symbol test variables between carnosine supplementation and placebo.

Stroop test variables	Placebo			Carnosine			*P**		
	**Baseline**	**Follow-Up**	**Δ**	**Baseline**	**Follow-Up**	**Δ**		*P2 (Pooled)*	*P(adj)*
**Score**	73.17±9.02	76.33±4.55	3.17±5.49	67.00±16.57	77.10±16.34	10.1±12.53	0.23	0.19	0.64

(Δ): follow-up - baseline values.
All data are represented as Mean ± Standard Deviation.
* p-value for differences between groups estimated by independent samples t-test for change (Δ).
P2(Pooled): Pooled p-values estimated by ANCOVA after 5 imputations using predictive mean matching to replace missing data.
P(adj): Adjusted p-value for false discovery rate using Benjamini-Hochberg correction method.
value

Table S2.3 Prediabetes: Multivariate regression analysis for the differences in change of Digit Symbol test variables between carnosine and placebo supplementation adjusted for covariates.

Digit Symbol test variables	Models	Change (Δ)						
		β	95% CI	SE	R^2	*P*	*P2* (Pooled)	*P(adj)*
**Score**	Model 1	3.23	-6.24, 12.70	0.13	0.45	0.48	0.43	0.49
	Model 2	3.58	-5.93, 13.10	0.14	0.48	0.44	0.42	0.49
	Model 3	5.16	-5.15, 15.49	0.21	0.51	0.30	0.41	0.49

Model 1: Adjusted for baseline value
Model 2: Adjusted for baseline value, diabetic status
Model 3: Adjusted for baseline value, diabetic status, level of education and age.
* p-values are estimated by Analysis of Covariance (ANCOVA).
$P2_{(Pooled)}$: Pooled p-values estimated by ANCOVA after 5 imputations using predictive mean matching to replace missing data.
P(adj): Adjusted p-value for false discovery rate using Benjamini-Hochberg correction method.

Table S2.4 Diabetes: Multivariate regression analysis for the differences in change of Digit Symbol test variables between carnosine and placebo supplementation adjusted for covariates.

Digit Symbol test variables	Models	Change (Δ)						
		β	95% CI	SE	R^2	*P*	*P2* (Pooled)	*P(adj)*
**Score**	Model 1	4.86	-6.29, 16.01	5.16	0.29	0.36	0.34	0.49

	Model 2	3.68	-8.18, 15.53	5.44	0.33	0.51	0.47	0.49
	Model 3	3.7	-8.77,16.18	5.67	0.33	0.53	0.49	0.49

Model 1: Adjusted for baseline value
Model 2: Adjusted for baseline value, diabetic status
Model 3: Adjusted for baseline value, diabetic status, level of education and age.
* p-values are estimated by Analysis of Covariance (ANCOVA).
P2(Pooled): Pooled p-values estimated by ANCOVA after 5 imputations using predictive mean matching to replace missing data.
P(adj): Adjusted p-value for false discovery rate using Benjamini-Hochberg correction method.

Table S3:Suplementary Stroop Tests

Table S3.1: STROOP test variables between carnosine supplementation and placebo.

**Stroop test variables**	**Placebo**			**Carnosine**			***P****	
	**Baseline**	**Follow-Up**	**Δ**	**Baseline**	**Follow-Up**	**Δ**		P(adj)
**Off Time (s)**	66.81 ± 7.90	64.22 ± 10.44	-2.45 ± 12.87	70.14 ± 13.62	72.24 ±10.01	0.56 ± 7.84	0.05	0.75
**Total number of runs (Stroop Off)**	5.69 ± 0.87	5.53 ±1.36	-0.067 ± 1.33	5.53 ± 0.64	5.54 ± 0.66	-0.77 ± 0.95	0.99	0.99
**Total number of runs (Stroop On)**	5.81 ± 1.42	5.87 ± 0.99	0 ± 1.41	5.67 ± 0.82	6 ± 1.08	0.31 ± 1.11	0.74	0.83

(Δ): follow-up - baseline values.
All data are represented as Mean ± Standard Deviation.
* p-value for differences between groups estimated by independent samples t-test for change **(Δ).**
P(adj): Adjusted p-value for false discovery rate using Benjamini-Hochberg correction method.
value

Table S3.2: Multivariate regression analysis for the differences in change of STROOP test variables between carnosine and placebo supplementation adjusted for covariates.

STROOP test variables	Models	Change (Δ)						
		β	95% CI	SE	R^2	*P**	*P2 (Pooled)*	P(adj)
**Total number of runs (Stroop Off)**	Model 1	-0.02	-0.90, 0.85	0.43	0.19	0.96	1.00	1.00
	Model 2	-0.05	-0.95, 0.85	0.43	0.20	0.91	0.95	0.99
	Model 3	-0.17	-1.12, 0.77	0.46	0.23	0.71	0.94	1.00
	Model 4	-0.07	-1.06, 0.92	0.48	0.25	0.88	0.86	0.97
**Total number of runs (Stroop On)**	Model 1	0.22	-0.58, 1.02	0.39	0.44	0.58	0.71	0.96
	Model 2	0.20	-0.62, 1.02	0.40	0.44	0.62	0.75	0.97
	Model 3	-0.07	-0.87, 0.73	0.39	0.55	0.86	0.85	0.98
	Model 4	--0.22	-1.04, 0.60	0.39	0.58	0.58	0.95	0.99

Model 1: Adjusted for baseline value
Model 2: Adjusted for baseline value, diabetic status
Model 3: Adjusted for baseline value, diabetic status and level of education
Model 4: Adjusted for baseline value, diabetic status, level of education and age.
* p-values are estimated by Analysis of Covariance (ANCOVA).
P2(Pooled): Pooled p-values estimated by ANCOVA after 5 imputations using predictive mean matching to replace missing data.
P(adj): Adjusted p-value for false discovery rate using Benjamini-Hochberg correction method.

(β) Unstandardized beta-coefficient
(CI) confidence interval
(SE) standard error, and
(R^2) R-square value

Table S3.3: Subgroup analysis

Table S3.3.1 Prediabetes: STROOP test variables between carnosine supplementation and placebo.

Stroop test variables	Placebo			Carnosine			*P**	P(adj)
	Baseline	Follow-Up	Δ	Baseline	Follow-Up	Δ		
**Off Time (s)**	67.74 ± 9.66	62.23 ± 12.66	-5.4 ± 16.95	73.03 ± 13.18	75.05±11.30	-2.27 ± 2.65	0.67	0.90
**On Time(s)**	74.723 ± 9.36	82.59 ± 24.92	7.86 ± 23.42	88.052 ±18.92	98.25 ± 25.97	10.19 ± 27.36	0.85	0.91
**Off Time + On Time(s)**	142.46 ± 18.26	180.56 ± 130.24	38.10 ± 130.64	161.078 ± 31.56	214.22 ±101.19	53.14 ± 111.41	0.80	0.90
**On time – Off time(s)**	6.99 ± 5.30	12.96 ± 19.31	5.52 ± 19.57	15.03 ± 8.22	12.77 ± 4.00	-2.00 ± 6.560	0.39	0.73
**Successful times x attempts (Off)**	377.512 ± 86.49	329.47 ± 122.49	-34.88 ± 150.34	414.24 ± 113.48	437.55 ± 84.07	-14.52 ± 92.29	0.78	0.90
**Successful times x attempts (On)**	455.93 ± 125.24	423.49 ± 85.94	-44.26 ± 112.73	470.05±98.02	505.21±128.93	37.87 ± 88.31	0.16	0.64
**Total number of runs (Stroop Off)**	5.56 ± .88	5.13 ± 1.46	-0.25 ± 1.67	5.13 ± 1.46	5.83 ± 0.75	-2.7756e-17 ± 1.095	0.76	0.90
**Total number of runs (Stroop On)**	6.11 ± 1.62	5.63 ± .74	-0.63 ±1.506	5.50 ± 0.76	6.17 ±1.47	0.67 ± 0.816	0.08	0.48

(Δ): follow-up - baseline values.
All data are represented as Mean ± Standard Deviation.
* p-value for differences between groups estimated by independent samples t-test for change **(Δ).**
P(adj): Adjusted p-value for false discovery rate using Benjamini-Hochberg correction method.
value

S3.3.2 <u>Diabetes</u>: Multivariate regression analysis for the differences in change of STROOP test variables between carnosine and placebo supplementation adjusted for covariates.

Stroop test variables	Placebo			Carnosine			*P**	*P(adj)*
	Baseline	Follow-Up	Δ	Baseline	Follow-Up	Δ		
**Off Time (s)**	66.3 ± 5.56	66.0 ± 8.05	-0.303 ± 4.6	66.83 ± 14.36	69.83 ± 8.91	2.99 ± 10.10	0.49	0.87
**On Time(s)**	78.68 ± 8.13	82.18 ± 11.28	3.50 ± 7.74	78.05 ± 11.46	80.67 ± 12.52	2.63 ± 7.08	0.73	0.90
**Off Time + On Time(s)**	144.95 ± 12.100	148.15 ± 19.104	3.20 ± 11.39	144.88 ± 24.97	150.50 ± 21.13	5.62 ± 15.10	0.82	0.90
**On time – Off time(s)**	12.40 ± 6.91	16.20 ± 4.38	3.80 ± 5.65	11.214 ± 7.19	10.84 ± 5.06	-0.37 ± 8.73	0.28	0.64
**Successful times x attempts (Off)**	396.52 ± 57.97	405.57 ± 84.60	9.05 ± 64.46	361.59 ± 74.51	366.96 ± 36.83	5.36 ± 62.49	0.91	0.92
**Successful times x attempts (On)**	432.47 ± 102.85	505.44 ± 116.46	9.05 ± 64.46	452.44 ± 63.59	473.18 ± 93.17	5.36 ± 62.49	0.29	0.64
**Total number of runs (Stroop Off)**	6.00 ± 0.89	6.17 ± 1.17	0.17 ± 0.98	5.43 ± 0.54	5.29 ± 0.49	-0.14 ± 0.90	0.56	0.90
**Total number of runs (Stroop On)**	5.50 ± 1.23	6.17 ± 1.33	0.67 ± 1.03	5.86 ± 0.90	5.86 ± 0.69	0 ± 1.29	0.30	0.64

(Δ): follow-up - baseline values.
All data are represented as Mean ± Standard Deviation.
[*] p-value for differences between groups estimated by independent samples t-test for change **(Δ).**
P(adj): Adjusted p-value for false discovery rate using Benjamini-Hochberg correction method.
value

Table S3.3.3 <u>Prediabetes</u>: Multivariate regression analysis for the differences in change of STROOP test variables between carnosine and placebo supplementation adjusted for covariates.

STROOP test variables	Models	Change (Δ)						
		β	95% CI	SE	R^2	*P*	*P2* *(Pooled)*	*P(adj)*
**Off Time**	Model 1	8.94	-1.30, 0.09	7.09	0.26	0.23	0.07	0.41
	Model 2	8.83	-8.45, 26.11	7.75	0.26	0.28	0.07	0.41
	Model 3	7.72	-12.28, 27.72	8.84	0.27	0.41	0.26	0.41

Model 1: Adjusted for baseline value

Model 2: Adjusted for baseline value, diabetic status
Model 3: Adjusted for baseline value, diabetic status, level of education and age.
* p-values are estimated by Analysis of Covariance (ANCOVA).
$P2_{(Pooled)}$: Pooled p-values estimated by ANCOVA after 5 imputations using predictive mean matching to replace missing data.
P(adj): Adjusted p-value for false discovery rate using Benjamini-Hochberg correction method.

(β) Unstandardized beta-coefficient
(CI) confidence interval
(SE) standard error, and
(R^2) R-square value

S3.3.4 Diabetes: Multivariate regression analysis for the differences in change of STROOP test variables between carnosine and placebo supplementation adjusted for covariates.

STROOP test variables	Models	Change (Δ)						
		β	95% CI	SE	R^2	*P*	*P2 (Pooled)*	*P(adj)*
**Off Time**	Model 1	2.50	-5.32, 10.33	3.56	0.37	0.50	0.48	0.5
	Model 2	3.12	-5.25, 11.48	3.75	0.40	0.43	0.41	0.5
	Model 3	3.18	-5.46, 11.82	3.82	0.44	0.43	0.41	0.5

Model 1: Adjusted for baseline value
Model 2: Adjusted for baseline value, diabetic status
Model 3: Adjusted for baseline value, diabetic status, level of education and age.
* p-values are estimated by Analysis of Covariance (ANCOVA).
$P2_{(Pooled)}$: Pooled p-values estimated by ANCOVA after 5 imputations using predictive mean matching to replace missing data.
P(adj): Adjusted p-value for false discovery rate using Benjamini-Hochberg correction method.

(β) Unstandardized beta-coefficient
(CI) confidence interval
(SE) standard error, and
(R^2) R-square value

Table S4: Trail Making Test subgroup analysis

Table S4.1 Prediabetes: Trail Making Test variables between carnosine supplementation and placebo.

Trail Making Test variables	Placebo			Carnosine			*P**	P(adj)
	Baseline	Follow-Up	Δ	Baseline	Follow-Up	Δ		
**Trail making test A (seconds)**	17.5±5.24	18.54±4.47	1.04±5.59	16.01±5.30	16.16±4.17	0.14±4.34	0.69	0.90
**Trail making test B (seconds)**	59.24±37.28	71.29±40.44	11.08±23.99	65.96±45.13	50.33±9.81	-15.62±46.28	0.09	0.48
**B:A (seconds)**	3.56±1.92	4.02±2.37	0.36±1.65	4.01±1.94	3.27 ±0.94	-0.74±2	0.18	0.64

(Δ): follow-up - baseline values.
All data are represented as Mean ± Standard Deviation.
* p-value for differences between groups estimated by independent samples t-test for change **(Δ).**
P(adj): Adjusted p-value for false discovery rate using Benjamini-Hochberg correction method.
value

Table S4.2 Diabetes: Trail Making Test test variables between carnosine supplementation and placebo.

Trail Making Test variables	Placebo			Carnosine			*P**	P(adj)
	Baseline	Follow-Up	Δ	Baseline	Follow-Up	Δ		
**Trail making test A (seconds)**	14.6±3.66	16.08±4.79	1.48±2.97	17.36±4.73	15.26±5.51	-2.10±6.68	0.24	0.64
**Trail making test B (seconds)**	54.32±14.02	46.33±16.68	-7.98±17	60.91±36.50	43.98±11.44	-16.93±41.15	0.62	0.90
**B:A (seconds)**	3.78±0.70	3±1.09	-0.78±1.25	3.56±1.98	3.01±0.66	-0.55±1.84	0.77	0.90

(Δ): follow-up - baseline values.
All data are represented as Mean ± Standard Deviation.
* p-value for differences between groups estimated by independent samples t-test for change **(Δ).**
P(adj): Adjusted p-value for false discovery rate using Benjamini-Hochberg correction method.
value

S4.3 Prediabetes: Multivariate regression analysis for the differences in change of STROOP test variables between carnosine and placebo supplementation adjusted for covariates.

Trail Making Test variables	Models	Change (Δ)						
		β	95% CI	SE	R^2	*P**	*P2 (Pooled)*	P(adj)
**Trail making test A (seconds)**	Model 1	-1.85	-5.41, 1.72	1.71	0.43	0.29	0.24	0.31
	Model 2	-1.83	-5.45, 1.80	1.74	0.44	0.31	0.19	0.31
	Model 3	-3.2	-6.34, -0.07	1.5	0.64	0.05	0.02	0.16
**Trail making test B (seconds)**	Model 1	-22.96	-47.93, 2	11.93	0.49	0.07	0.06	0.16
	Model 2	-23.28	-48.54, 1.98	12.02	0.51	0.07	0.06	0.16
	Model 3	-24.69	-51.05, 1.68	12.5	0.52	0.07	0.06	0.16
**B:A (seconds)**	Model 1	-.954	-2.469, 0.56	0.72	0.26	0.20	0.24	0.31
	Model 2	-.964	-2.51, 0.59	0.74	0.27	0.21	0.25	0.31
	Model 3	-.905	-2.54, 0.73	0.78	0.28	0.26	0.3	0.31

Model 1: Adjusted for baseline value
Model 2: Adjusted for baseline value, diabetic status
Model 3: Adjusted for baseline value, diabetic status, level of education and age.
* p-values are estimated by Analysis of Covariance (ANCOVA).
P2(Pooled): Pooled p-values estimated by ANCOVA after 5 imputations using predictive mean matching to replace missing data.
P(adj): Adjusted p-value for false discovery rate using Benjamini-Hochberg correction method.

S4.4 Diabetes: Multivariate regression analysis for the differences in change of STROOP test variables between carnosine and placebo supplementation adjusted for covariates.

STROOP test variables	Models	Change (Δ)						
		β	95% CI	SE	R^2	*P**	*P2 (Pooled)*	P(adj)

**Trail making test A (seconds)**	Model 1	-1.86	-7.78, 4.07	2.76	0.3	0.51	0.5	0.92
	Model 2	-1.28	-7.49, 4.94	2.88	0.34	0.67	0.66	0.92
	Model 3	-1.41	-7.91, 5.10	2.99	0.35	0.65	0.64	0.92
**Trail making test B (seconds)**	Model 1	-2.04	-17.15, 13.08	7.05	0.86	0.78	0.77	0.92
	Model 2	-0.89	-16.88, 15.1	7.4	0.86	0.91	0.9	0.92
	Model 3	-1	-16.75, 14.74	7.23	0.88	0.89	0.89	0.92
**B:A (seconds)**	Model 1	0.04	-0.86, 0.95	0.42	0.77	0.92	0.92	0.92
	Model 2	0.08	-0.9, 1.07	0.45	0.77	0.86	0.85	0.92
	Model 3	0.06	-0.93, 1.05	0.45	0.79	0.89	0.89	0.92

Model 1: Adjusted for baseline value
Model 2: Adjusted for baseline value, diabetic status
Model 3: Adjusted for baseline value, diabetic status, level of education and age.
* p-values are estimated by Analysis of Covariance (ANCOVA).
$P2_{(Pooled)}$: Pooled p-values estimated by ANCOVA after 5 imputations using predictive mean matching to replace missing data.
P(adj): Adjusted p-value for false discovery rate using Benjamini-Hochberg correction method.

Table S5.1: Supplementary CANTAB tests results for differences between carnosine supplementation and placebo.

**CANTAB test variables**	**Placebo (n=19)**			**Carnosine (n=20)**			P^*	
	**Baseline**	**Follow-Up**	**Δ**	**Baseline**	**Follow-Up**	**Δ**		$\mathbf{P_{(adj)}}$
DMS Percent Correct (0 Second Delay)	92.00±10.05	84±17	-8.00±24.62	89.52±22.47	90±15	0.48±28.98	0.35	0.70
DMS Percent Correct (4 Second Delay)	87.00±14.90	90±15	4.00±16.67	80.95±18.41	85±18	3.81±27.29	0.98	1.00
DMS Percent Correct (12 Second Delay)	75.00±21.40	87±17	12.00±26.28	81.90±15.37	81±15	-0.95±23.22	0.10	0.46
PALTA 4 (Paired Associates Learning Total Attempts 4 Patterns)	1.58±0.61	1.24±0.70	-0.32±.89	1.25±0.72	1.45±0.83	0.21±0.71	0.05	0.59
PALTEA 8 (PAL Total errors 8 shapes adjusted)	10.05±8.38	8.66±8.41	-1.37±6.12	12.8±9.11	8.85±6.86	-3.79±9.29	0.35	0.67
SWMBE4 (SWM Between Errors 4 Boxes)	0.80±0.95	0.71±1.38	-0.05±1.36	0.57±1.25	0.33±0.73	-0.24±1.18	0.64	0.84

SWMBE6 (Spatial Working Memory - between errors 6 boxes)	3.30±3.90	3.50±3.22	0.20±3.81	3.81±3.17	2.00±2.68	-1.81±3.27	0.08	0.53
SWMBE8 (Spatial Working Memory - between errors 8 boxes)	7.70±6.43	8.10±6.89	0.40±7.37	7.95±5.31	7.10±5.97	-0.86±6.94	0.58	0.83

(Δ): follow-up - baseline values.
All data are represented as Mean ± Standard Deviation.
* p-value for differences between groups estimated by independent samples t-test for change **(Δ).**
P(adj): Adjusted p-value for false discovery rate using Benjamini-Hochberg correction method.
value

Table S5.2: Subgroup analysis
Table S5.2.1 Prediabetes: CANTAB test variables between carnosine supplementation and placebo.

CANTAB test variables	Placebo			Carnosine			P^*	P(adj)
	Baseline	Follow-Up	Δ	Baseline	Follow-Up	Δ		
PALFAMS (Paired Associates Learning First attempt memory score)	12.53 ± 3.04	14.87 ± 3.04	2.33 ± 2.41	12.56 ± 4.19	10.22 ± 4.06	-3.00 ± 3.70	0.001	0.03
PALMETS (Paired Associates Learning Mean Errors to Success)	2.13 ± 1.46	1.13 ± 0.74	-1.00 ± 1.46	1.44 ± 1.88	2.78 ± 1.39	1.38 ± 1.60	0.002	0.03
PALTA (Paired Associates Learning Total Attempts)	8.47 ± 1.85	6.93 ± 1.87	-1.53 ± 1.36	8.00 ± 2.12	8.67 ± 1.58	0.63 ± 2.50	0.013	0.14
PRMPCD (Pattern Recognition Memory Percent Correct Delayed)	95.24 ± 7.10	93.33 ± 7.84	-2.38 ± 8.29	85.83 ± 14.19	81.67 ± 14.06	-4.16 ± 19.35	0.76	0.90

(Δ): follow-up - baseline values.
All data are represented as Mean ± Standard Deviation.
* p-value for differences between groups estimated by independent samples t-test for change **(Δ).**

P(adj): Adjusted p-value for false discovery rate using Benjamini-Hochberg correction method.
value

Table S5.2.2 <u>Prediabetes:</u> Multivariate regression analysis for the differences in change of CANTAB test variables between carnosine and placebo supplementation adjusted for covariates.

CANTAB tests	Models	Change (Δ)						
		β	95% CI	SE	R^2	*P*	*P2* (pooled)	*P(adj)*
**PALFAMS (Paired Associates Learning First attempt memory score)**	Model 1	-5.30	-7.68 , -2.92	1.14	0.58	<0.001	<0.001	0.58
	Model 2	-5.31	-7.76 , -2.856	1.17	0.58	<0.001	<0.001	0.49
	Model 3	-5.25	-7.846 , -2.650	1.24	0.58	<0.001	<0.001	0.48
**PALMETS (Paired Associates Learning Mean Errors to Success)**	Model 1	1.92	1.037, 2.803	0.42	0.77	<0.001	<0.001	0.18
	Model 2	1.92	1.033, 2.811	0.43	0.78	<0.001	<0.001	0.18
	Model 3	1.90	0.952, 2.855	0.45	0.78	<0.001	<0.001	0.18
**PALTA (Paired Associates Learning Total Attempts)**	Model 1	1.92	0.494, 3.35	0.68	0.49	0.011	0.006	0.48
	Model 2	1.98	0.706, 3.25	0.61	0.62	0.004	0.003	0.36
	Model 3	1.94	0.574, 3.298	0.65	0.62	0.008	0.005	0.36
**PRMPCD (Pattern Recognition Memory Percent Correct Delayed)**	Model 1	-9.67	-20.05, 0.72	4.99	0.41	0.07	0.032	0.49
	Model 2	-9.97	-19.98, 0.03	4.80	0.48	0.05	0.023	0.40
	Model 3	-9.87	-20.19, 0.45	4.93	0.48	0.06	0.029	0.40
**SWMS (Spatial Working Memory Strategey)**	Model 1	-0.06	-1.82, 2.14	.794	-0.09	0.94	0.94	1.0
	Model 2	-0.05	-1.74, 1.63	.810	-0.13	0.95	0.95	1.0
	Model 3	-0.02	-1.86, 1.53	.814	-0.13	0.84	0.84	1.0
	Model 1	-2.22	-9.31, 4.86	3.42	0.12	0.52	0.52	0.84
	Model 2	-2.03	-8.38. 4.33	3.06	0.29	0.51	0.51	0.84

**SWMBE (Spatial Working Memory – Between Errors)**	Model 3	-2.25	-8.8, 4.31	3.14	0.27	0.48	0.48	0.83
**SWMBE6 (Spatial Working Memory Between Errors – 6 trials)**	Model 1	-0.58	-2.98, 1.8	1.15	0.26	0.62	0.61	0.99
	Model 2	-0.52	-2.64,1.6	1.02	0.42	0.62	0.61	0.99
	Model 3	-0.63	-2.79, 1.54	1.04	0.41	0.55	0.55	0.84

Model 1: Adjusted for baseline value
Model 2: Adjusted for baseline value, diabetic status
Model 3: Adjusted for baseline value, diabetic status, level of education and age.
* p-values are estimated by Analysis of Covariance (ANCOVA).
$P2_{(Pooled)}$: Pooled p-values estimated by ANCOVA after 5 imputations using predictive mean matching to replace missing data.
P(adj): Adjusted p-value for false discovery rate using Benjamini-Hochberg correction method.

(β) Unstandardized beta-coefficient
(CI) confidence interval
(SE) standard error, and
(R^2) R-square value

Table S5.2.3 Diabetes: CANTAB test variables between carnosine supplementation and placebo.

CANTAB test variables	Placebo (n=)			Carnosine ()			P^*	*P(adj)*
	**Baseline**	**Follow-Up**	**Δ**	**Baseline**	**Follow-Up**	**Δ**		
PALFAMS (Paired Associates Learning First attempt memory score)	11.00 ± 2.83	13.00 ± 5.22	1.25 ± 5.91	12.82 ± 4.14	14.36 ± 2.84	1.55 ± 4.74	0.92	0.92
PALMETS (Paired Associates Learning Mean Errors to Success)	1.75 ± 1.50	1.50 ± 1.52	-1.00 ± 1.83	1.55 ± 1.29	2.00 ± 1.26	0.45 ± 1.04	0.07	0.14

PALTA (Paired Associates Learning Total Attempts)	7.75 ± 1.50	6.67 ± 1.63	-1.50 ± 2.08	7.36 ± 1.75	6.82 ± 1.25	-0.55 ± 1.69	0.38	0.51
PRMPCD (Pattern Recognition Memory Percent Correct Delayed)	93.75 ± 7.98	93.33 ± 14.91	8.33 ± 8.34	88.33 ± 9.78	87.12 ± 13.62	-2.50 ± 15.74	0.29	0.46

(Δ): follow-up - baseline values.
All data are represented as Mean ± Standard Deviation.
* p-value for differences between groups estimated by independent samples t-test for change **(Δ).**
P(adj): Adjusted p-value for false discovery rate using Benjamini-Hochberg correction method.
value

Table S5.2.4 Diabetes: Multivariate regression analysis for the differences in change of CANTAB test variables between carnosine and placebo supplementation adjusted for covariates.

**CANTAB tests**	**Models**	**Change (Δ)**						
		β	95% CI	SE	R^2	*P*	*P2* *(pooled)*	*P (adj)*
**PALFAMS (Paired Associates Learning First attempt memory score)**	Model 1	1.78	-3.52, 7.09	2.43	0.40	0.48	0.59	0.9
	Model 2	1.79	-4.01, 7.59	2.63	0.40	0.51	0.62	1.00
	Model 3	1.72	-4.47, 7.90	2.78	0.40	0.55	0.48	0.80
**PALMETS (Paired Associates Learning Mean Errors to Success)**	Model 1	1.34	-0.03, 2.71	0.63	0.49	0.05	0.31	0.61
	Model 2	1.50	0.07, 2.93	0.65	0.53	0.04	0.24	0.6
	Model 3	1.55	0.20, 2.89	0.60	0.63	0.03	0.27	0.6
	Model 1	0.66	-1.00, 2.32	0.76	0.54	0.40	0.67	0.97
	Model 2	0.98	-0.62, 2.58	0.73	0.64	0.21	0.38	0.78

**PALTA (Paired Associates Learning Total Attempts)**	Model 3	1.09	0.08, 2.10	0.45	0.87	0.04	0.41	0.79
**PRMPCD (Pattern Recognition Memory Percent Correct Delayed)**	Model 1	-13.66	-32.74, 5.43	8.56	0.38	0.14	0.46	0.79
	Model 2	-15.91	-35.58, 3.77	8.70	0.45	0.10	0.24	0.6
	Model 3	-15.35	-36.55, 5.85	9.19	0.46	0.13	0.19	0.58
**SWMS (Spatial Working Memory Strategey)**	Model 1	-0.98	-4.49, 2.54	1.63	0.38	0.56	0.55	0.84
	Model 2	-1.17	-4.93, 2.59	1.73	0.34	0.51	0.5	0.83
	Model 3	-0.79	-4.4, 2.65	1.57	0.46	0.62	0.44	0.77
**SWMBE (Spatial Working Memory – Between Errors)**	Model 1	-5.72	-14.92, 3.48	4.26	0.31	0.20	0.18	0.58
	Model 2	-6.14	-15.96, 3.68	4.51	0.26	0.20	0.17	0.58
	Model 3	-5.29	-14.95, 4.37	4.39	0.31	0.25	0.15	0.57
**SWMBE6 (Spatial Working Memory Between Errors – 6 trials)**	Model 1	-2.65	-5.46. 0.15	1.30	0.61	0.06	0.02	0.36
	Model 2	-2.46	-5.43, 0.51	1.36	0.01	0.1	0.04	0.48
	Model 3	-2.40	-5.51, 0.75	1.43	0.003	0.12	0.05	0.49

Model 1: Adjusted for baseline value

Model 2: Adjusted for baseline value, diabetic status

Model 3: Adjusted for baseline value, diabetic status, level of education and age.

* p-values are estimated by Analysis of Covariance (ANCOVA).

$P2_{(Pooled)}$: Pooled p-values estimated by ANCOVA after 5 imputations using predictive mean matching to replace missing data.

P(adj): Adjusted p-value for false discovery rate using Benjamini-Hochberg correction method.

(β) Unstandardized beta-coefficient
(CI) confidence interval
(SE) standard error, and
(R^2) R-square valu
